# Novel genome characteristics contribute to the invasiveness of *Phragmites australis* (common reed)

**DOI:** 10.1111/mec.16293

**Published:** 2021-12-11

**Authors:** Dong‐Ha Oh, Kurt P. Kowalski, Quynh N. Quach, Chathura Wijesinghege, Philippa Tanford, Maheshi Dassanayake, Keith Clay

**Affiliations:** ^1^ Department of Biological Sciences Louisiana State University Baton Rouge Louisiana USA; ^2^ U.S. Geological Survey Great Lakes Science Center Ann Arbor Michigan USA; ^3^ Department of Ecology & Evolutionary Biology Tulane University New Orleans Louisiana USA; ^4^ Department of Biology Washington University in St. Louis St. Louis Missouri USA; ^5^ Department of Biology Indiana University Bloomington Indiana USA

**Keywords:** Arundinoideae, fungal inoculation, gene expression, invasive, *Phragmites australis*, reference genome, whole genome duplication

## Abstract

The rapid invasion of the non‐native *Phragmites australis* (Poaceae, subfamily Arundinoideae) is a major threat to native wetland ecosystems in North America and elsewhere. We describe the first reference genome for *P*. *australis* and compare invasive (ssp. *australis*) and native (ssp. *americanus*) genotypes collected from replicated populations across the Laurentian Great Lakes to deduce genomic bases driving its invasive success. Here, we report novel genomic features including a *Phragmites* lineage‐specific whole genome duplication, followed by gene loss and preferential retention of genes associated with transcription factors and regulatory functions in the remaining duplicates. Comparative transcriptomic analyses revealed that genes associated with biotic stress and defence responses were expressed at a higher basal level in invasive genotypes, but native genotypes showed a stronger induction of defence responses when challenged by a fungal endophyte. The reference genome and transcriptomes, combined with previous ecological and environmental data, add to our understanding of mechanisms leading to invasiveness and support the development of novel, genomics‐assisted management approaches for invasive *Phragmites*.

## 1 INTRODUCTION

Invasion of native ecosystems by non‐native species is a worldwide problem that damages both ecosystems and economies. Invasive plants can negatively affect agricultural production (Dogra et al., 2010; Pimentel et al., 2005) and displace native species through multiple mechanisms including enemy release (Keane & Crawley, 2002; Mitchell & Power, 2003), allelopathy (Chengxu et al., 2011; Kalisz et al., 2020), and novel traits (Divíšek et al., 2018; Kolar & Lodge, 2001). They are key drivers of global environmental change and require substantial economic resources for management (Diagne et al., 2021). Given the biological impacts of invasive species, there is an urgent need for studies across diverse systems to evaluate the long‐term consequences of invasive species and to better understand the underlying biological mechanisms of invasion. Dozens of hypotheses have been proposed to explain why some species become invasive (Catford et al., 2009) and why some habitats are vulnerable to invasion (Stohlgren et al., 2002), which can contribute to better management of invasive species and a better understanding of general mechanisms of invasiveness (Hierro et al., 2005). The genetic and molecular bases for evolution of invasive lineages have been of longstanding interest, but there is still limited information addressing this problem (Baker & Stebbins, 1965; Bock et al., 2015). Previous studies have examined the genetics of invasive and weedy species independent of direct comparisons with noninvasive genotypes or populations (Liu, Yan, et al., 2020; Peng et al., 2014), but better insights can be gained from comparative studies where invasive and noninvasive genotypes or populations of the same species are investigated in both the native and invasive ranges (Hodgins et al., 2013; Lavergne & Molofsky, 2007; Lockwood & Somero, 2011; Stern & Lee, 2020) or where invasive and noninvasive populations occur intermixed in the same habitats (Mounger et al., 2021; Sherman et al., 2016). Understanding the evolutionary bases of invasiveness will contribute to the development of more effective management and control strategies.

**FIGURE 1:**
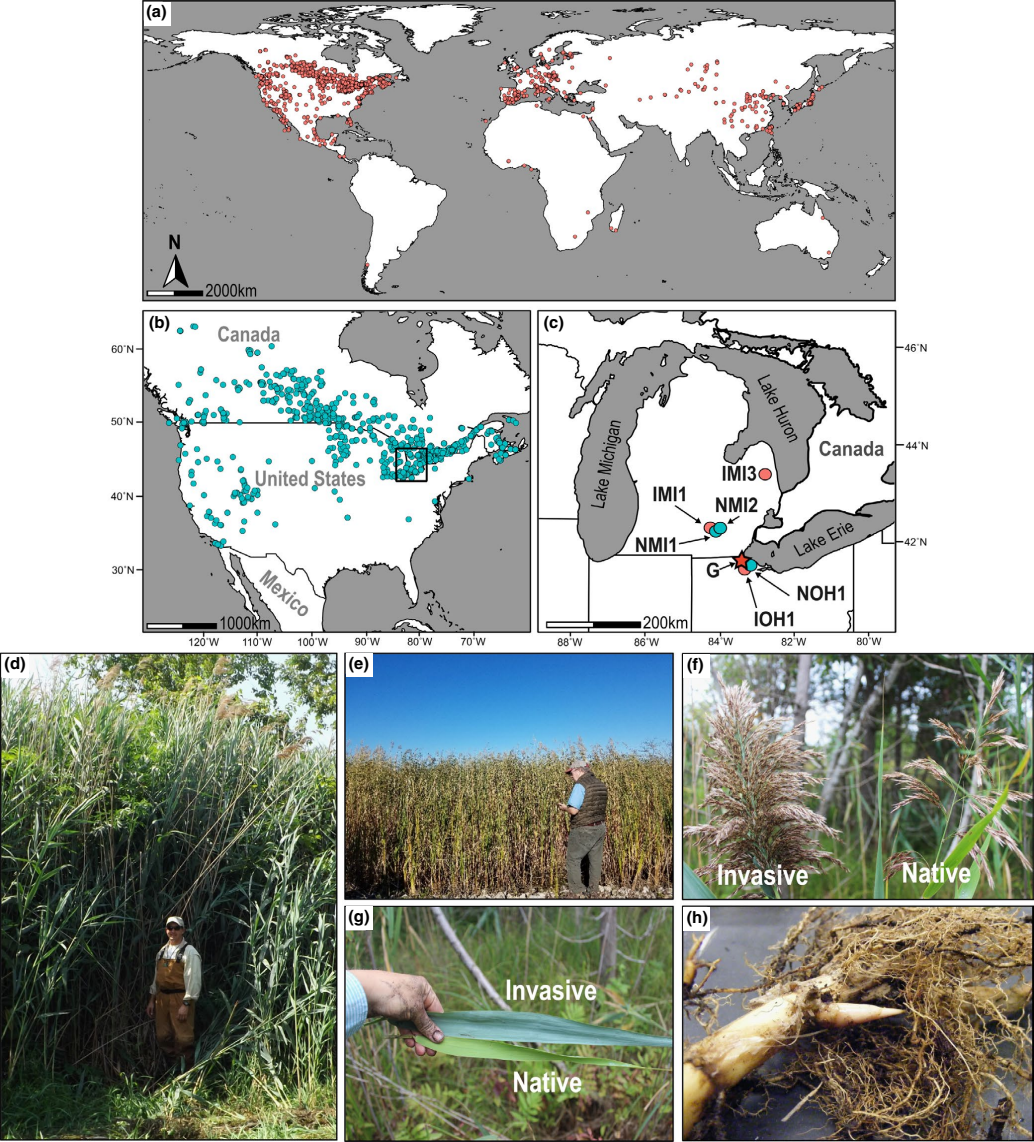
The invasive and native subspecies of common reed (*Phragmites australis*). (a) Reported global distribution of *Phragmites australis* ssp. *australis* (“Invasive”) and (b) Reported distribution of *P*. *australis* ssp. *americanus* (“Native”) in the U.S.A. and Canada based on the Global Biodiversity Information Facility (GBIF.org, 2020a, 2020b). (c) A magnified view of the box in panel b showing sample site locations in Michigan (MI) and Ohio (OH) in the U.S.A. Invasive (I) and Native (N) *Phragmites* plants were sampled from three Michigan sites (MI1, MI2, and MI3) and one Ohio site (OH1) in the Great Lakes region. Clonal fragments for the reference genome (G) were collected near the OH1 site. See Figure 5a for more details. (d) Invasive *P*. *australis* ssp. *australis* stand growing in a western Lake Erie coastal wetland. (e) Native *P*. *australis* ssp. *americanus* stand located in a western Lake Erie wetland. (f) Seed heads from the invasive and native *P*. *australis* growing in Michigan. (g) Invasive and native *P. australis* leaves collected in Michigan. (h) Rhizome and dense fibrous roots from an invasive *P. australis* plant growing in Michigan

*Phragmites australis* (Cav.) Trin. ex Steud. (Common Reed, Poaceae) is globally distributed (Figure 1a) and provides multiple ecosystem services in its native range (Kiviat, 2013; Rooth & Stevenson, 2000; Whitaker et al., 2015). A native subspecies (*P*. *australis* ssp. *americanus*) has been present in North American wetlands for thousands of years (Saltonstall et al., 2004). However, the non‐native, invasive subspecies (*P*. *australis* ssp. *australis*) (Martin & Blossey, 2013; Saltonstall, 2002) was introduced to North America from Europe prior to 1900 and has been aggressively disrupting and displacing native plant communities (Mozdzer et al., 2013; Saltonstall, 2002) and altering wildlife habitat and ecosystem properties (Perez et al., 2013; Rogalski & Skelly, 2012). The invasive subspecies occurs throughout the contiguous United States (U.S.) and the entire Laurentian Great Lakes basin (Bourgeau‐Chavez et al., 2013; Saltonstall, 2002; Tulbure & Johnston, 2010) (Figure 1b) and is one of the most problematic invasive plant species in wetland habitats in eastern North America, with millions of dollars per year invested in control efforts (Kowalski et al., 2015; Meyerson et al., 2016). It co‐occurs with the native subspecies in many areas (Figure 1c) but exhibits more robust growth (Figure 1d,e) with larger inflorescences, leaves, and height (Figure 1f,g). Both native and invasive subspecies reproduce by seed and clonally via rhizomes (Figure 1h). A variety of mechanisms promoting *P*. *australis* invasions have been proposed, including efficient resource utilization and lower construction costs (Caplan et al., 2014), genetic diversity and mode of reproduction (Kettenring et al., 2011; Kettenring & Mock, 2012), and escape from natural enemies relative to native *Phragmites* genotypes (Allen et al., 2015; Cronin et al., 2015; Lambert et al., 2007), but effective control strategies are lacking (Hazelton et al., 2014). The Great Lakes Restoration Initiative has identified invasive species (including *P*. *australis)* as one of its five most urgent issues since 2010 (Great Lake Restoration Initiative, 2019). Invasive *P*. *australis* has also been recognized as the leading plant model for studying genetic mechanisms underlying plant invasions (Cesarino et al., 2020; Meyerson et al., 2016). *P. australis* therefore provides an excellent system to test genetic adaptations and control measures in plant invasions, given that both native and invasive populations coexist over a large geographic range (Figure 1a, b).

*Phragmites australis* exhibits a range of ploidy levels from 2 to 12× and higher, with tetraploids reported as dominant in temperate Europe and North America and octoploids dominant in Asia (Clevering & Lissner, 1999; Saltonstall, 2003; te Beest et al., 2012). *P. australis* genotypes, ploidy levels, and genome size have been assessed for traits that may favour invasiveness such as photosynthetic rate and nitrogen use efficiency, rhizome size, shoot emergence rate, and herbivory resistance (Guo et al., 2014; Park & Blossey, 2008; Pyšek et al., 2020). However, *P*. *australis* has not been investigated from a genomic perspective and lacks a reference genome that can serve as a foundational resource to investigate genomic traits underlying plant invasions and to identify genetic targets for biocontrol. Considering the diverse range of ploidy levels (Keller, 2000; Liu, Yin, et al., 2020; Pyšek et al., 2020) and that the genetic mechanisms and natural selection underlying diploidization of polyploids are largely unknown for angiosperms (Li et al., 2021), *P*. *australis* provides an ideal subject to study such evolutionary mechanisms. Further, *Phragmites* belongs to the grass subfamily Arundinoideae, which has been poorly explored at the genomic level compared to other grass subfamilies, even though multiple species are ecologically dominant or invasive on a global scale (Grass Phylogeny Working Group, 2001).

We report here the first reference genome for *P. australis* using a representative genotype from the invasive subspecies *P*. *australis* ssp. *australis*, as well as comparative transcriptomic analyses of invasive and native genotypes coexisting in the Great Lakes region of North America. Our results identify variation in gene expression correlated with invasiveness and provide a key genomic resource for grasses and the subfamily Arundinoideae, novel insights into evolution in an underexplored grass clade, and a genomic foundation for development of new management approaches.

## 2 MATERIALS AND METHODS

### 2.1 Plant materials

For genome sequencing, tillers and associated rhizome tissues were collected from a single *P*. *australis* clump of chloroplast haplotype M (Martin & Blossey, 2013; Saltonstall, 2002) at the Ottawa National Wildlife Refuge near Toledo, Ohio (Figure 1c, marked with “G”) and propagated in a walk‐in growth chamber as detailed in Supporting Information Methods. For transcriptome analyses, we collected three additional invasive and three native genotypes from four sites around the Great Lakes in Michigan and Ohio, U.S. (Figure 1c). Native genotypes were readily distinguished from invasive genotypes by their smaller stature and thinner tillers with distinctive reddish coloration at the nodes (Figure 1e, f, g). Specimens of both subspecies are in many Midwestern herbaria (https://midwestherbaria.org/portal/), and voucher specimens generated from the genomic reference have been deposited in the Indiana University, Louisiana State University, and University of Michigan herbaria. Both native and invasive genotypes were confirmed by chloroplast haplotype sequences as previously described (Saltonstall, 2002, 2016). *Phragmites* plants were grown in the growth chamber and subjected to endophyte inoculation treatments before RNA isolation for RNA‐seq analyses, as detailed in Supporting Information Methods.

### 2.2 Genome sequencing, assembly, and annotation

For the genome sequencing, genomic DNA was isolated from leaf tissue of the individual sample collected (“G” in Figure 1c) using a 2% CTAB extraction protocol (Doyle & Dickson, 1987), converted to SMRTbell libraries (Pacific Bioscience) with 20‐Kb target insert size, and sequenced for continuous long reads (CLR) using a PacBio RSII single‐molecule real‐time sequencing platform. After post‐processing (SMRT Analysis v2.3, Pacific Bioscience), 4.79 million reads (mean length 8.80 Kbp, N50 13.11 Kbp, and total 42.19 Gbp) were assembled into contigs using Canu assembler (v. 1.4) (Koren et al., 2017), with the target genome size set to 1 Gbp, generating the reference genome assembly (Supporting Information Methods). In addition, the same genomic DNA sample was converted to TruSeq DNA paired‐ends libraries (Illumina) for whole‐genome shotgun sequencing on a NextSeq platform (Illumina) and used to test for functional diploidy based on sequence diversity sampled (Supporting Information Methods and Figure S1).

For genome annotation, we first used RepeatModeler v. 1.0.8 and RepeatMasker v. 4.0.9 (http://www.repeatmasker.org/) to detect transposable elements and repetitive sequences in the assembled *P*. *australis* contigs. De novo‐detected repetitive sequences were combined with known monocot repeat sequences in RepBase (v. 23.07; http://www.girinst.org/) to mask repeats in *P*. *australis* contigs. Protein‐coding gene models on the repeat‐masked genome were predicted using the MAKER (v. 2.31.10) (Campbell et al., 2014) with *P*. *australis* benchmarking universal single‐copy orthologues (BUSCO)‐trained parameters for ab initio gene model prediction (Waterhouse et al., 2018) and hints from *P*. *australis* transcriptomes de novo assembled using Trinity (v. 2.1.1) (Haas et al., 2013) as well as from homologues from *Sorghum* (Phytozome ID:454) and *Brachypodium* (Phytozome ID:314). In addition, we performed a reference‐guided transcriptome assembly using StringTie (v. 2.0.1) (Pertea et al., 2015) to report putative isoform models associated with gene models predicted in the reference genome. Transcriptomes used to assist the protein‐coding gene model prediction of the reference genome were assembled based on paired‐end RNA‐seq reads derived from the leaf, shoot, and rhizome tissues of the same sample used for the reference genome assembly. The gene model encoding the longest open reading frame (ORF) was selected as the representative for each of the observed protein‐coding gene loci.

### 2.3 Comparative and functional analyses

We used BUSCO (v. 3) (Waterhouse et al., 2018) to assess the completeness of the representative *P*. *australis* protein‐coding gene models in comparison with grass genomes available in Monocots PLAZA (v. 4.5) (Van Bel et al., 2018) (Table S1). Maximum‐likelihood species trees were created using OrthoFinder (v. 2.2.7) (Emms & Kelly, 2019) and RAxML (v. 8.2) (Stamatakis, 2006) based on a concatenated alignment of 782 protein‐coding sequences. We selected five monocot genomes for in‐depth comparative analyses, based on their availability of recently updated gene models with >90% BUSCO scores. These genomes lack additional whole genome duplication (WGD) events more recent than the ρ WGD event documented for grasses (McKain et al., 2016). MCscan (as implemented in JCVI v. 1.1.7) (Tang et al., 2008) was used to compare syntenic depths between *P*. *australis* and other monocot genomes. SynMap (Lyons & Freeling, 2008) and CLfinder pipeline (Oh & Dassanayake, 2019) were used to detect colinear paralogue and orthologue pairs within the *P*. *australis* genome and between *P*. *australis* and monocot genomes, respectively. We estimated synonymous substitution rates at four‐fold degenerate sites using codeml (Yang, 1997) as described previously (Oh & Dassanayake, 2019). We detected orthologue groups among representative gene models of *P*. *australis* and other monocot genomes using OrthoFinder (v. 2.2.7) (Emms & Kelly, 2019) and MMseqs2 (Steinegger & Söding, 2017) and subsequently identified *P*. *australis* orthologue groups that are “conserved” and “duplicated” in orthologue copy numbers compared to other monocot species as detailed in Supporting Information Methods.

Gene ontology (GO) annotations were transferred to the *Phragmites* representative gene models based on sequence similarities to plant proteins with GO annotations as of 1 January, 2020 in the GO consortium (http://geneontology.org/). In short, *Phragmites* protein sequences were compared with reference sequences with a GO annotation using MMseqs2 with the maximum sensitivity (‐s 7.5). If the protein alignment covers minimum 30% of both the query and subject sequences, the GO annotation was transferred to the *Phragmites* protein. We used BiNGO (Maere et al., 2005) to detect GO terms enriched in *Phragmites* gene models. GO terms were further clustered and summarized using GOMCL (Wang et al., 2020).

### 2.4 Endophyte treatment, transcriptome assembly, and RNA‐seq analyses

Invasive and native genotypes of *P*. *australis* were propagated from rhizome cuttings, grown in a growth chamber for 60 days, and subjected to *Alternaria alternata* (accession KT923239) (Clay et al., 2016) fungal endophyte inoculation. *A. alternata* was used for inoculations because it was widespread across all regions sampled and was the second most common endophyte isolated from invasive *Phragmites* leaf tissues (Clay et al., 2016). The endophyte has also been shown to enhance allelopathic effects of host plants (Aschehoug et al., 2014). RNA for control and endophyte‐treated samples were extracted from mature leaf and rhizome tissue separately as eight biological replicates (four plants, sampled twice per plant) per sample for six genotypes (three native and three invasive genotypes) and used to generate 192 RNAseq libraries following Illumina TruSeq library preparation guidelines as described in Supporting Information Methods. RNA‐seq reads were filtered for adapter sequences using FASTP (Chen et al., 2018) and aligned to the reference genome using HISAT2 (v. 2.2). Expression counts for each gene model were estimated using StringTie (v. 2.0.1) with default parameters (Pertea et al., 2016). RNA‐Seq reads were mapped to protein‐coding gene models in the reference genome using bowtie2 (v. 2.4.4) with the “‐‐very‐sensitive” option to assess the rate of mapped reads among transcriptome samples. Differentially expressed genes (DEGs) were identified based on a minimum 2‐fold difference in expression levels, with adjusted *p*‐values < .05 estimated by DESeq2 (Love et al., 2014), between pairs of genotypes or between endophyte‐inoculated and control samples within each genotype. In addition, putative protein‐coding transcript sequences were obtained from filtered RNA‐Seq reads by the Trinity (v. 2.1.1) and TransDecoder (v. 5.5.0) pipeline (Haas et al., 2013) (Table S2) and used for estimating the phylogenetic relationship of *P*. *australis* subspecies and genotypes using the Agalma pipeline (v. 2), which was designed to work with orthologous gene alignments derived from both genomes and transcriptomes (Dunn et al., 2013).

## 3 RESULTS

### 3.1 Assembly and annotation of the common reed *Phragmites australis*

We sequenced the genome of an invasive genotype of *Phragmites australis* ssp. *australis* collected in the U.S. Fish and Wildlife Service, Ottawa National Wildlife Refuge, Ohio (Figure 1c, star‐marked “G”). We obtained ~42.19 Gbp of high confidence sequence data from leaf genomic DNA, representing a 37‐fold genome coverage, using PacBio SMRT sequencing technology. Assembled using Canu (Koren et al., 2017) (version 1.4; see Methods), the reference genome provides 1.14 Gbp of 13,411 gap‐free contigs with more than half of the assembled genome captured in 1370 contigs (N50) larger than 194.6 kbp (L50). The largest contig was 3.22 Mbp (Table 1). Illumina short reads mapped to the primary assembly confirmed the genotype used as the reference to be functionally diploid based on single nucleotide polymorphisms that represented a nonreference allele frequency distribution with a peak at 0.5, as expected with a functionally diploid genome (Figure S1). In total, 56.19% of the genome consisted of repetitive sequences, with sequences derived from long terminal repeat (LTR) retrotransposons constituting 36.42% of the genome (Table S3). Based on the repeat‐masked genome sequence, we annotated 64,857 protein‐coding gene models with a total length of 72.35 Mbp, which accounted for 6.35% of the genome (Table 1). The genomic location of these gene models and their best orthologues in rice and *Arabidopsis thaliana* are provided in Supporting Information data set 1. BUSCO analysis (Waterhouse et al., 2018) found 93.3% of single‐copy gene models expected for land plants in the *Phragmites* reference genome (Table S1).

**TABLE 1:** The *Phragmites australis* draft genome

Assembly	
Assembled genome size	1,139,927,050 bps
Largest contig size	3,219,705 bps
N50 contig length (L50)	194,574 bps
Total number of contigs	13,411
Number of contigs >1 Mbps	67
Number of contigs >N50	1370
Annotation	
Number of protein‐coding gene loci	64,857
Total length of predicted ORFs	72,347,598 bps
Longest ORF	14,790 bps
Median length of ORFs	927 bps
Proportion of ORFs in the genome	6.35%
Proportion of repeats in the genome	56.19%
SINEs	0.14%
LINEs	1.74%
LTR elements	36.42%
DNA elements	11.43%
Unclassified repeats	6.46%

**FIGURE 2:**
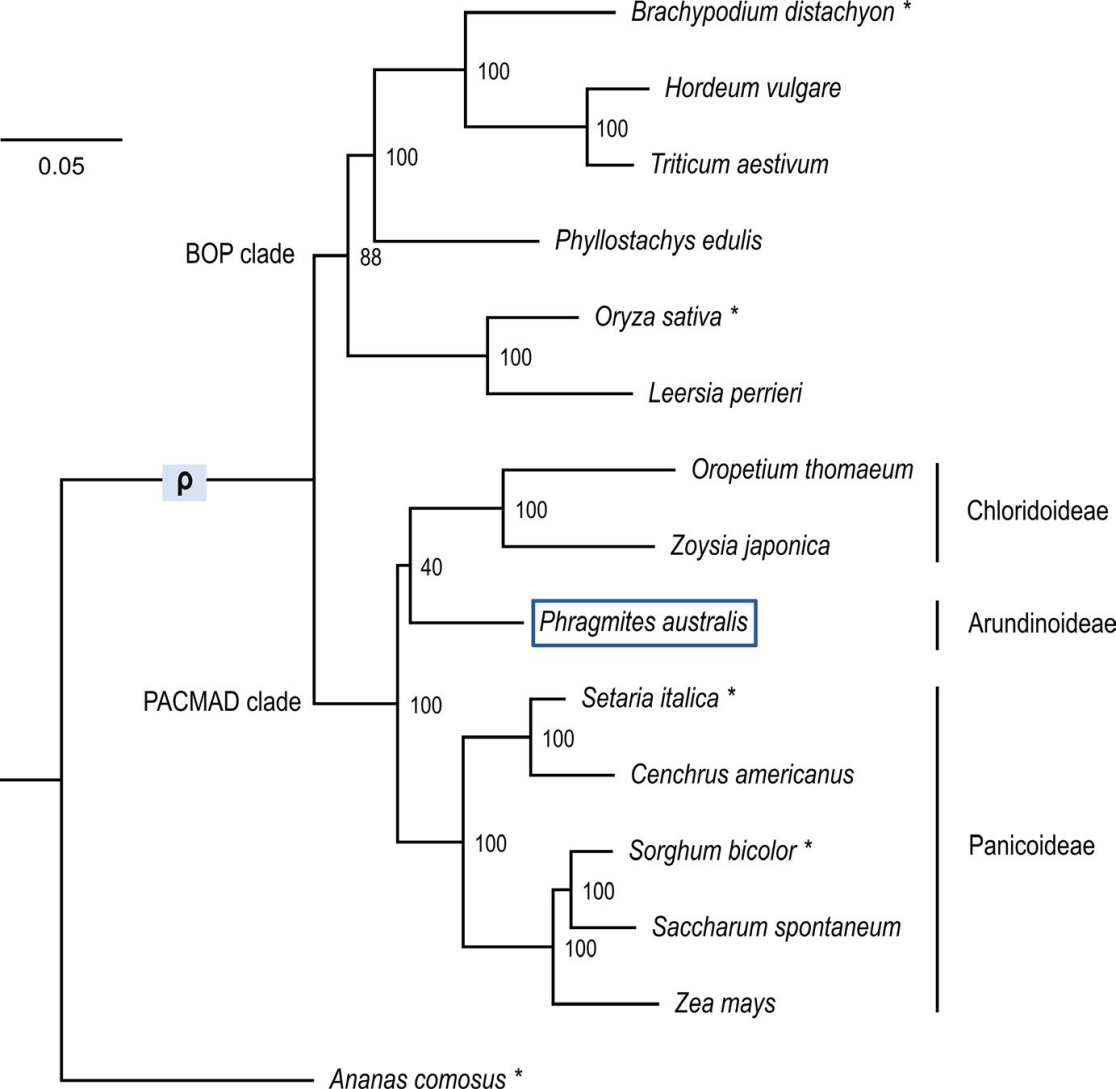
Phylogenetic position of *Phragmites australis* in the underinvestigated Arundinoideae subfamily, based on the draft genome. For the *P*. *australis* draft genome and 14 monocot genomes publicly available in Monocots PLAZA (v. 4.5) (Van Bel et al., 2018), a maximum likelihood species tree was constructed based on 26,878 amino acid alignments from 782 orthologue groups, selected based on the criteria that at least seven species among the set had a single orthologue. All sites that included gaps in more than 20% of taxa were excluded. The number in each branch shows percent support from 1000 bootstrap replicates. The branch with the ρ genome duplication (McKain et al., 2016) is marked, and species with asterisks were used for comparative analyses

We constructed a species tree using 6404 gene models representing 782 gene families from *P*. *australis* and 13 other published grass genomes (PLAZA monocot database v. 4.5) (Van Bel et al., 2018), with pineapple (*Ananas comosus*, Bromeliaceae) as the outgroup. *Phragmites australis*, representing the first genome from the Arundinoideae subfamily, was placed sister to subfamily Chloridoideae, consistent with the PACMAD clade species tree (Figure 2) (Burke et al., 2016; Hardion et al., 2017).

### 3.2 Signatures of a whole genome duplication event in the lineage of *P. australis*

**FIGURE 3:**
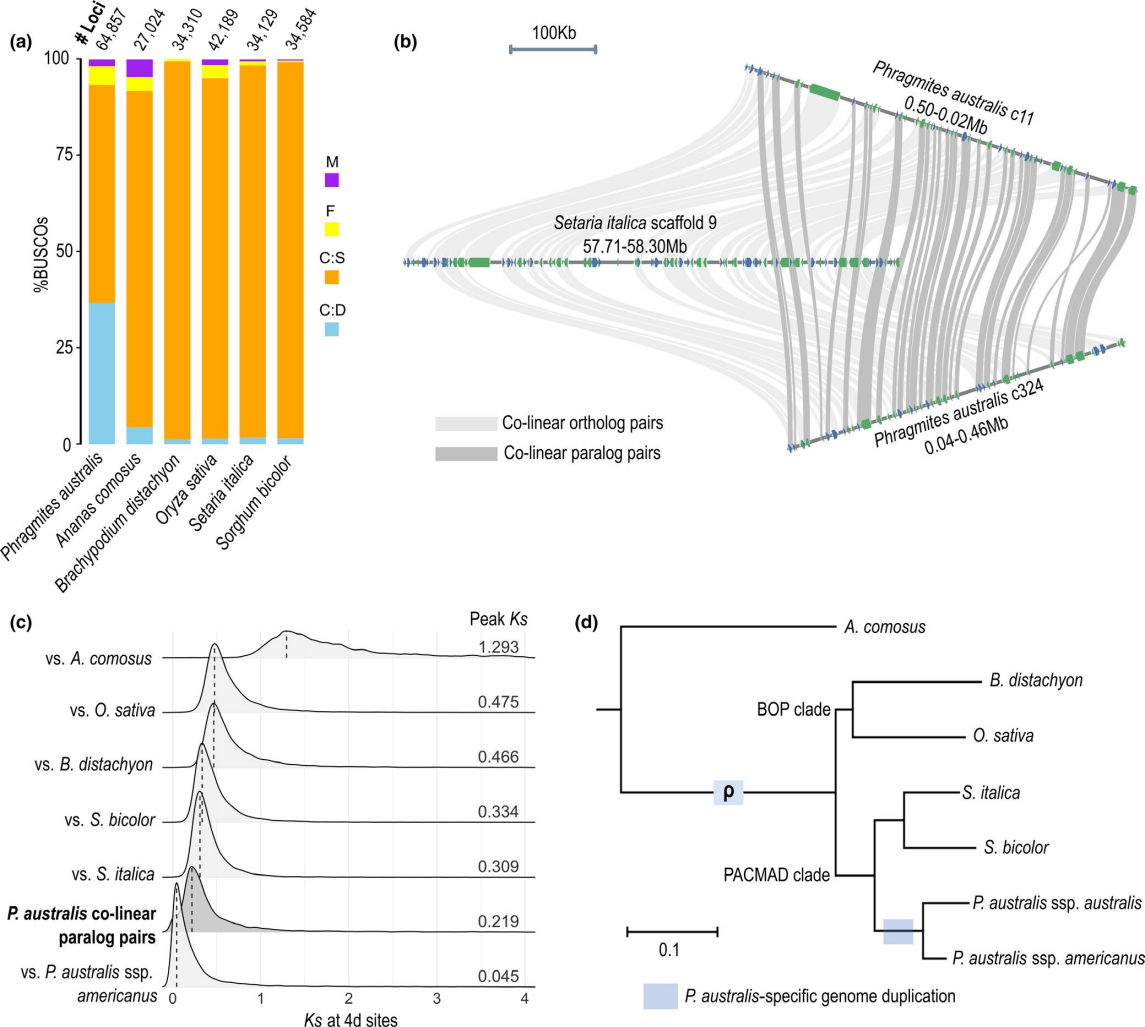
Signatures of *Phragmites australis*‐specific whole genome duplication (WGD). (a) Percentages of complete duplicated (C:D), complete single‐copy (C:S), fragmented (F), and missing (M) orthologues among 1375 Benchmarking universal single‐copy orthologues (BUSCOs) and the number of protein‐coding gene loci (# loci), in the genomes of *P*. *australis* and other monocot species. (b) An example microsynteny between a 500‐kb *Setaria italica* genomic block and two duplicated *P*. *australis* genomic blocks. Ribbons connect colinear orthologue (light grey) and paralogue (dark grey) pairs identified by MCscan (Tang et al., 2008) as described in Methods. (c) Synonymous substitution rates (*Ks*) at four‐fold degenerate (4d) sites were estimated for colinear orthologue pairs between *P*. *australis* and *A*. *comosus* (total 2720 orthologue pairs), *B*. *distachyon* (10,559 pairs), *O. sativa* (11,176 pairs), *S. italica* (12,878 pairs), and *S. bicolor* (12,322 pairs), respectively, as well as 6600 colinear paralogue pairs detected within the *P*. *australis* genome. For comparison with the native *P*. *australis* ssp. *americanus*, we used 11,445 reciprocal best homologue pairs between *P*. *australis* reference genome and a de novo transcriptome assembly from *P*. *australis* ssp. *americanus*. Probability distributions and peak values of *Ks* are shown. (d) A maximum‐ likelihood species tree shows the branches associated with the *P*. *australis*‐specific WGD, as well as the ρ WGD event shared among grasses (McKain et al., 2016)

We found signatures of a previously unreported whole genome duplication (WGD) in the *P*. *australis* genome that occurred after its divergence from the subfamily Panicoideae (Figure 3a–d). This was detected as a more recent lineage specific event following the ρ WGD event identified for multiple lineages in Poales (McKain et al., 2016). We compared the *P*. *australis* genome with five representative monocot reference genomes, including pineapple (*A*. *comosus*) and four grass species that did not experience an additional WGD event following the ρ duplication (Figures 2 and 3d marked with asterisks). The *P*. *australis* genome presented 36.7% BUSCO genes as duplicated, while only 1%–5% BUSCOs were duplicated in the five comparator genomes (Figure 3a and Table S1). A genome‐wide alignment between *Setaria italica* and *P*. *australis* found that 50.2% of *S*. *italica* gene models are represented twice in the *P*. *australis* genome as colinear orthologues in synteny blocks (Figure 3b, and Figure S2a). Similar patterns were observed in comparisons between *P*. *australis* and the other four monocot genomes (Figure S2a). A genome‐wide self‐alignment using SynMap identified 14,005 gene loci, comprising 21.6% of all *P*. *australis* protein‐coding genes, that were organized into 1501 paralogous synteny blocks consisted of at least five colinear paralogue pairs. Synteny blocks were widespread across the *P*. *australis* genome and found in 66.8% of all contigs with 10 or more protein‐coding gene loci (Figures S2b,c), further supportive of a WGD event in the lineage leading to *P*. *australis*.

To further assess the timing of genome duplications that resulted in the observed paralogous synteny blocks, we used neutral evolutionary substitutions calculated for four‐fold degenerate (4D) sites in codons that allows positioning of timing of duplicated events within a clade. We plotted the distribution of synonymous substitution rate (*Ks*) at 4D sites for *P*. *australis* colinear paralogue pairs and for colinear orthologue pairs between *P*. *australis* and comparator species (Figure 3c). The peak *Ks* for *P*. *australis* colinear paralogue pairs (Figure 3c, dark grey) was smaller than those observed for any pairwise comparisons between species, while larger than the value found between invasive *P*. *australis* ssp. *australis* and native *P*. *australis* ssp. *americanus* (Figure 3c, bottom row). We also found a small proportion of synteny blocks within the *P*. *australis* genome that probably represent the ρ duplication at the root of the Poaceae (Figure S3). Our analysis suggests that the majority of colinear paralogues in synteny blocks are derived from a single WGD event that occurred after the divergence of *Phragmites* from the Panicoideae, represented here by *S*. *italica* and *S. bicolor*, but before the divergence between the subspecies *australis* and *americanus* (Figure 3d).

### 3.3 Biased retention of transcription factors and signalling‐related genes following the lineage‐specific genome duplication in *P. australis*

**FIGURE 4:**
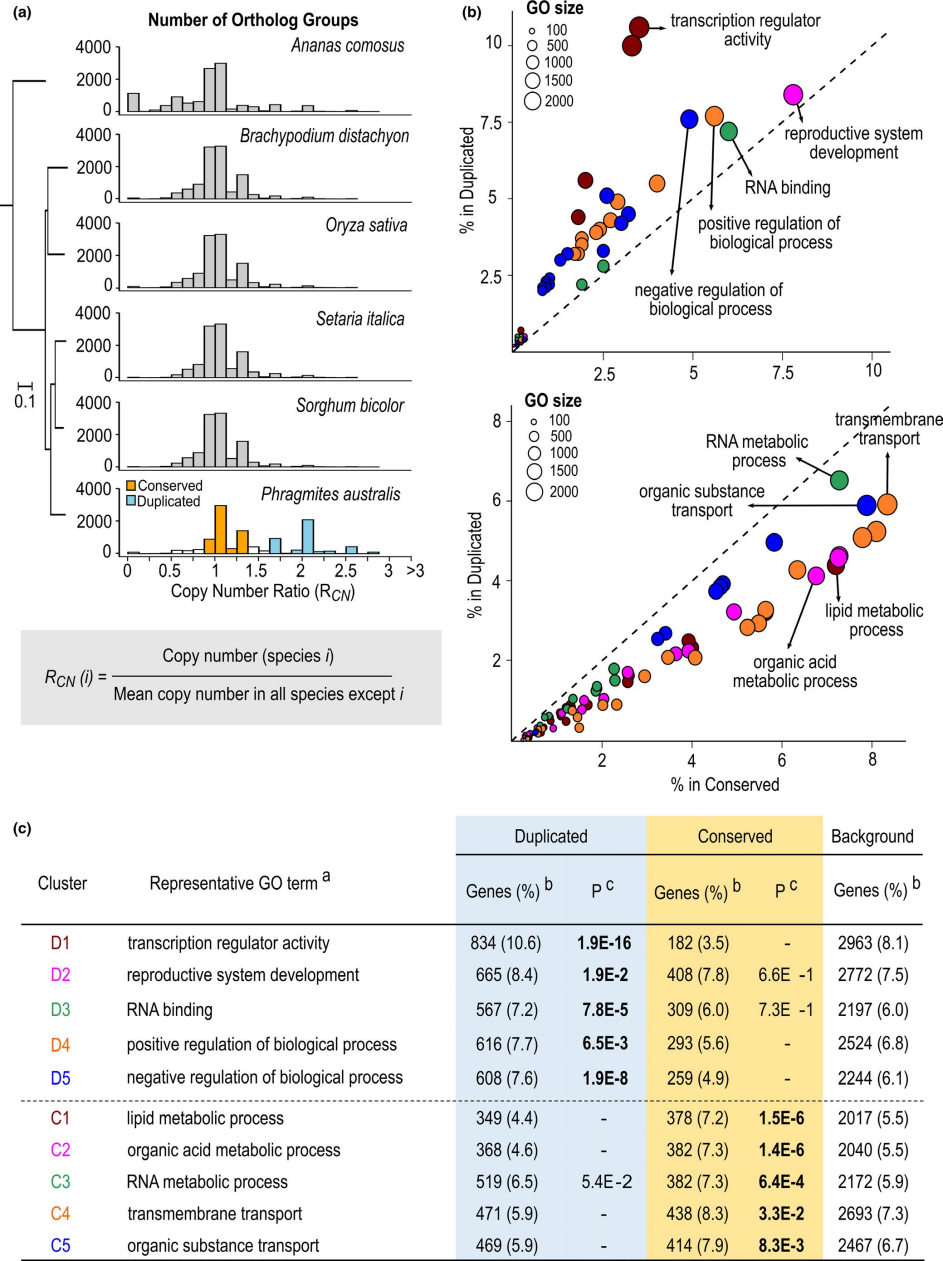
Functions enriched among *Phragmites australis* genes that remained duplicated after the WGD event. (a) Comparison of orthologue copy numbers between *P*. *australis* and five other monocot species. For each orthologue group identified by OrthoFinder as described in Methods, copy number ratio (*R*_CN_) was calculated for each species by dividing the orthologue number in the species with the mean orthologue copy number in the other five species. Histograms show a shift towards increased *R*_CN_ uniquely in *P*. *australis*. We identified “conserved” (orange) and “duplicated” (sky‐blue) groups among *P*. *australis* genes whose copy numbers remain unchanged and uniquely increased, respectively, compared to other monocot species. (b, c) GO terms enriched in either duplicated or conserved *P*. *australis* gene groups. The proportion of genes annotated with each GO term in duplicated and conserved groups is plotted as circles in (b). GO terms at least 80% overlapping with a bigger GO term are clustered and the largest five GO clusters enriched in either group were shown with the same colour in (b) and (c). ^a^The largest GO term in each GO cluster; MF, molecular function; BP, biological process. ^b^Percentages are the total number of genes with a GO annotation in each group. ^c^ *p*‐Values of enrichment compared to the background, after Benjamini‐Hochberg correction for multiple testing. Values <0.05 are in bold

While the synteny blocks are widespread across the *P*. *australis* genome, the relatively short length of each synteny block (Figure S2c) and the fact that only 21.6% of duplicated genes were retained in synteny blocks indicates a substantial fractionation following genome duplication, probably due to the selective retention of duplicates that enhance plant fitness while reducing nonessential duplicates or extra copies of genes under strong dosage‐dependent selection. We therefore compared the functions of duplicated *P*. *australis* genes relative to those genes with conserved copy numbers based on orthologue groups including other grass species (Figure 4).

For all orthologue groups (OGs) detected among *P*. *australis* and the five comparator monocot species (Methods and Supporting Information data set 2), we calculated the orthologue copy number ratio (*R*_CN_) for each species by dividing the copy number in the species with the average of the other five species (Figure 4a and Table S4). All species except *Phragmites* showed a peak at *R*_CN_ ≈ 1. By contrast, the *R*_CN_ distribution in *P*. *australis* showed two peaks, one with *R*_CN_ at 1 (Figure 4a, “conserved”) and a second peak at *R*_CN_ ≈ 2 (Figure 4a, “duplicated”), indicating that a substantial number of *P*. *australis* OGs have their copy numbers doubled compared to other species (see Methods for details). We searched for enriched gene functions in the duplicated group (11,002 *P*. *australis* genes in 4113 OGs) and in the Conserved group (6981 *P*. *australis* genes in 5600 OGs). Figure 4b, c present the five largest functional clusters, detected using GOMCL (Wang et al., 2020), considering GO terms significantly enriched exclusively in either conserved or duplicated groups. The largest clusters included 72% and 64% of all *Phragmites* genes represented by 123 and 146 GO terms enriched in the duplicated and the conserved groups, respectively (Supporting Information data set 3). Functions enriched exclusively in the duplicated group were largely related to regulation of gene expression (Figure 4b,c). For example, “transcription regulator activity” is the most enriched functional cluster among duplicated genes (Figure 4b,c, cluster D1), representing 10.6% of all genes in the duplicated group with a GO annotation, compared to only 3.5% and 8.1% in the conserved group and all genes (used as the background for the enrichment analysis), respectively (Supporting Information data set 3). By contrast, the conserved group was enriched in functions associated with primary metabolic processes and transport (C1–5 in Figure 4b, c and Supporting Information data set 3). Selective gene retention after WGD events are often linked to adaptive traits or traits leading to lineage diversification. The biased retention of transcription factors and regulatory processes in *P*. *australis* spp. *australis* following its most recent WGD event is indicative of greater genomic plasticity conducive to invasive lifestyles (Clark & Donoghue, 2018).

### 3.4 Divergence in basal transcriptome profiles between invasive and native *P*. *australis* subspecies

**FIGURE 5:**
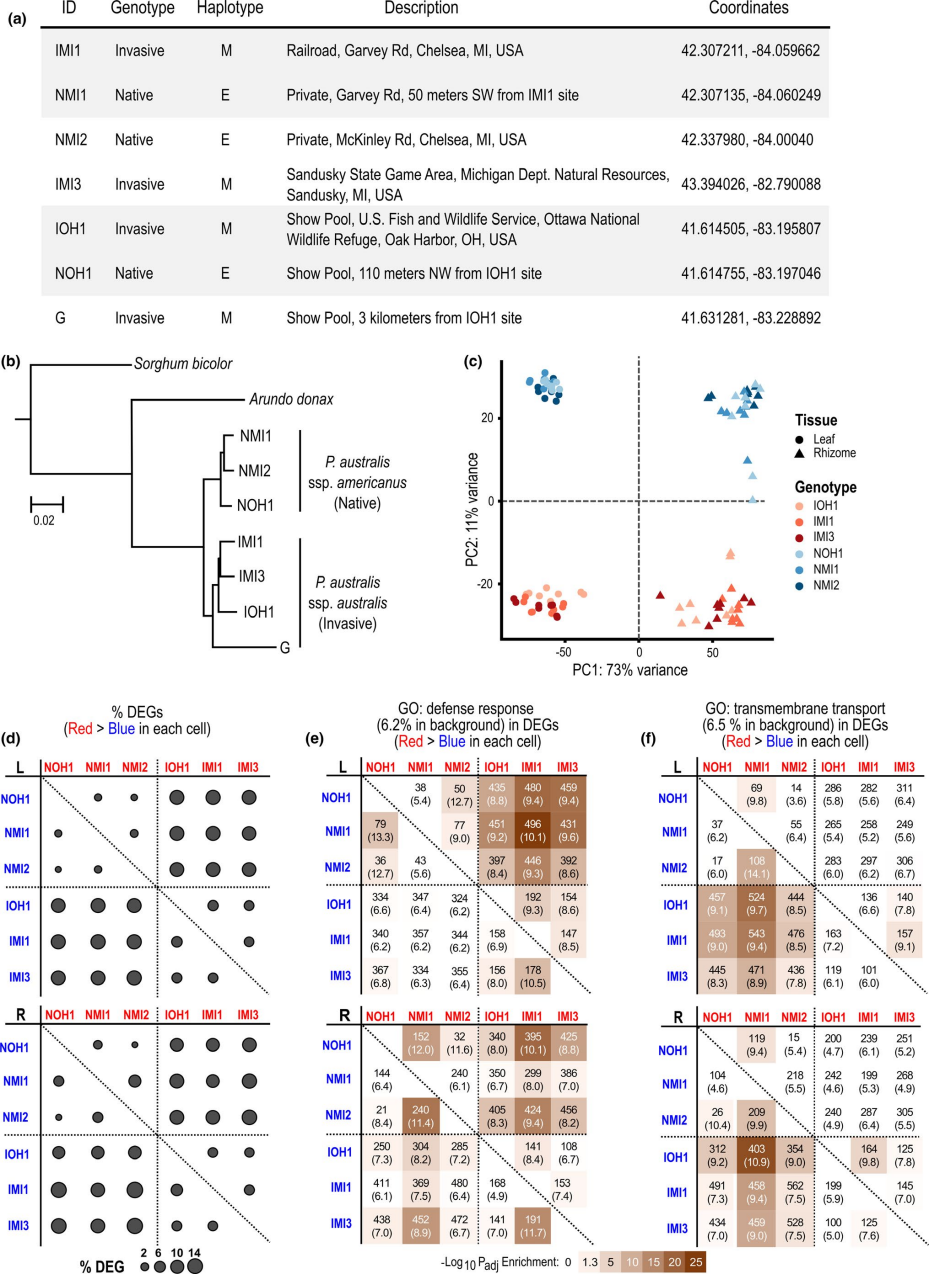
Comparison of transcriptomes between invasive and native *Phragmites australis* genotypes. (a) Details on genotype collections of invasive (I) and native (N) subspecies from locations marked on Figure 1c,b A maximum likelihood tree based on deduced protein sequences of 5394 homologous gene groups. Protein sequences were deduced from transcriptomes de novo assembled using RNA‐seq reads derived from the six *P*. *australis* genotypes shown in (a) as well as the reference gene models (marked with “G”). Publicly available transcriptome and genome sequences were used for *Arundo donax* (Barrero et al., 2015) and *Sorghum bicolor*, respectively. All branches were 100% supported by 100 bootstrap tests. (c) RNA‐seq reads from leaf and rhizome tissues of the six genotypes were aligned to the *P*. *australis* reference genome. Principal component analysis separated different tissues and genotypes. (d) Differentially expressed genes (DEGs) showing significant (adjusted *p*‐value <.05 and fold‐difference ≥2) changes in basal‐level expression in leaf (L) and rhizome (R) were identified between all pairs of genotypes. In the diagonal plot, the circle in each cell represents the proportion of DEGs among 64,857 *P*. *australis* reference gene models in which the genotype in the column (red) shows higher basal‐level expression than the clone in the row (blue). (e, f) GO terms “defence response” (e) and “transmembrane transport” (f) showed enrichment among DEGs in which invasive and native genotypes showed higher basal‐level expression in pairwise comparisons, respectively. Each cell shows the number and percentage of DEGs annotated with the GO term among all DEGs showing higher basal‐level expression in the genotype specified by the column (red) compared to the genotype by the row (blue). Adjusted *p*‐values of enrichment were calculated compared to a background of 41,595 reference gene models annotated with any GO term and represented as a colour heatmap

To gain further insight into genetic factors promoting invasiveness, we compared transcriptomes of invasive and native *Phragmites* subspecies collected from the Great Lakes region (Figures 1c and 5a). We generated eight replicates of RNA‐seq samples from leaf and rhizome tissues, respectively (see Methods), followed by independent de novo transcriptome assembly of the six genotypes (Table S2). The maximum likelihood tree based on concatenated alignments of 5394 homologue groups separated the six genotypes into invasive and native subspecies as expected, and the three invasive genotypes were placed together with the invasive genotype used as the reference genome (Figure 5b).

We aligned RNA‐seq reads to the reference genome and gene model sequences to explore the differences in basal transcriptome profiles between invasive and native genotypes and estimate the relative abundance of reference genes, as detailed in Methods (Supporting Information data set 4). Proportions of RNA‐seq reads aligned to protein‐coding gene model sequences did not show significant differences between invasive and native genotypes, while the native genotypes showed overall slightly fewer reads aligned to the reference genome sequences (Table S5). Further, when normalized to the total number of reads aligned to protein‐coding gene models and expressed gene compositions as implemented in DESeq2, the distribution of estimated expression values and number of significantly differentially expressed genes (DEGs) did not show a bias towards either invasive or native genotypes (Figure S4). The leaf‐ and rhizome‐derived transcriptomes were distinct from each other in a principal component analysis (PCA) (on PC1), while both showed additional separation between native and invasive genotypes (on PC2) (Figure 5c). In agreement with the PCA, the number of DEGs between invasive and native genotypes was much greater than DEGs detected between any other genotype comparisons (Figure 5d). On average, 11.5 ± 1.0% of the 64,857 reference gene models showed higher basal expression in the invasive genotypes compared to the native genotypes (Figure 5d, upper right sections of the diagonal plots). Similarly, 12.1 ± 2.1% of gene models were more highly expressed in the native genotypes than in the invasive genotypes (Figure 5d, lower left sections).

In the leaf samples, “response to stimulus” and “response to stress” were the largest representative GO terms showing significant enrichment among DEGs with higher basal expression in the invasive genotypes compared to the native genotypes in all cross‐genotype comparisons (Supporting Information data set 5). Interestingly, among child GO terms of these two GO terms, only those categorized as “response to biotic stimulus” and “defence response” showed significant enrichment in the invasive genotypes, but not “response to abiotic stress” (Figure S5). On average, 9.2% of all DEGs were annotated with “defence response” and showed higher basal expression in the invasive genotypes compared to 6.4% and 6.2% in the native genotypes and the background, respectively (Figure 5e, upper panel). This bias towards a higher basal expression of genes associated with biotic stress and defence in the invasive genotypes was less clear in the rhizome tissue (Figure 5e, lower panel). By contrast with the functional enrichment in defence responses observed for the invasive genotypes, the native genotypes were biased towards genes associated with “transmembrane transport” and its child GO terms, including “ammonium transport”, are represented by DEGs with higher basal expression in all cross‐genotype comparisons (Figure 5f and Supporting Information data set 5). This transcriptomic signal was more prominent in leaf samples compared to rhizomes as similarly observed for the defence response.

### 3.5 Divergence in invasive and native *P. australis* transcriptomic responses to biotic stress induced by fungal endophyte inoculation

**FIGURE 6:**
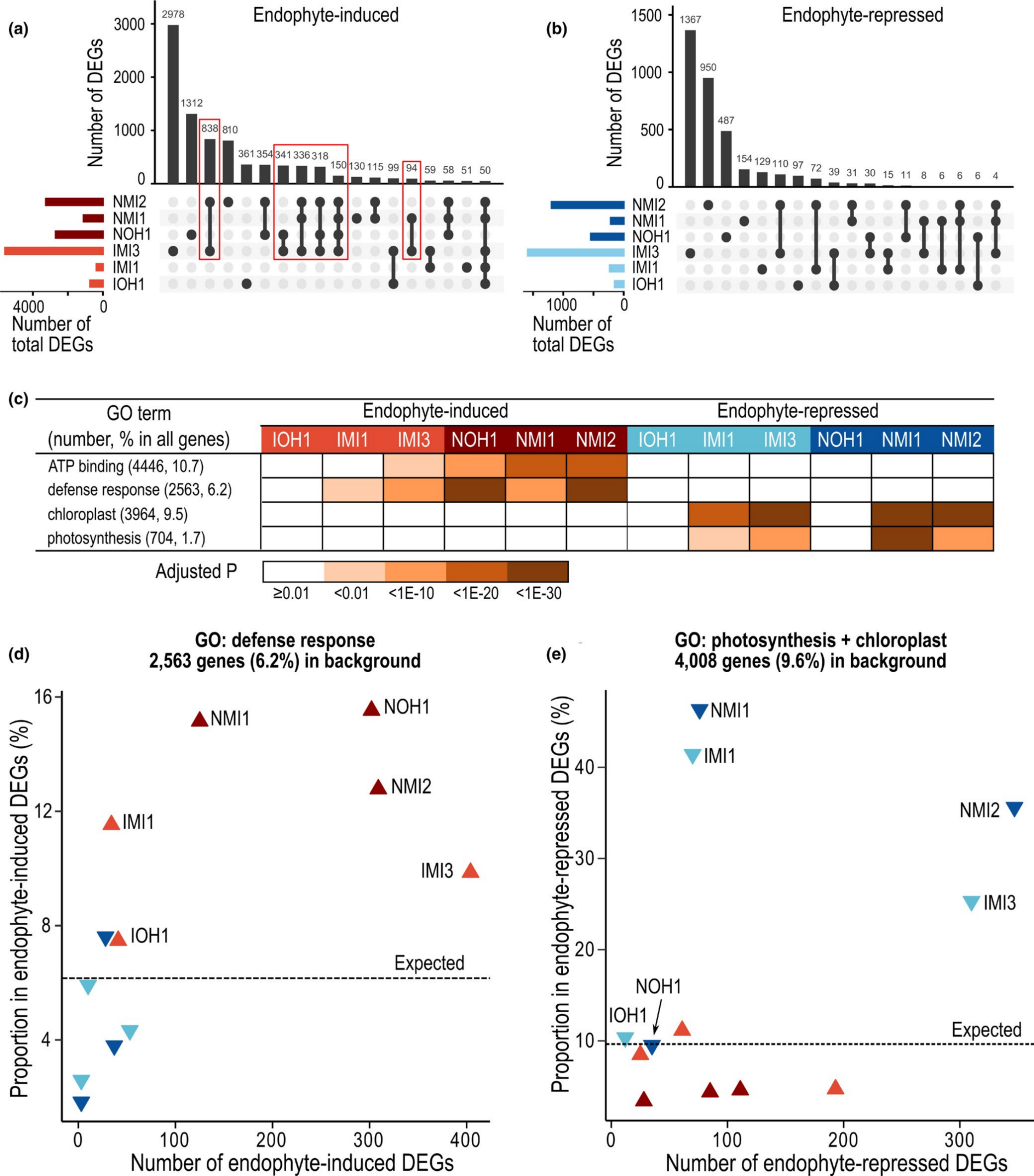
Transcriptome responses to *Alternaria alternata* fungal endophyte inoculation of invasive and native *Phragmites australis* genotypes. (a, b) Upset plots showing the number of shared and unique endophyte‐induced (a) and repressed (b) DEGs among the six invasive and native genotypes. Red boxes show endophyte‐induced DEGs shared between IMI3 genotype and a native genotype. (c) The pattern of GO enrichment in six clones involving the largest number of endophyte‐induced DEGs (represented by GO terms “ATP binding” and “defence response”) and endophyte‐repressed DEGs (represented by GO terms “chloroplast” and “photosynthesis”). (d, e) Number and percent proportion of endophyte‐induced (upward triangles) or repressed (downward triangles) DEGs annotated with the GO term “defence response” (d) or either of the GO terms “photosynthesis” and “chloroplast” (e). The percent proportions in the entire *P*. *australis* gene models are marked with dashed lines as the expected value when there is no enrichment

We inoculated target genotypes with the fungal endophyte commonly isolated from *P*. *australis*, *Alternaria alternata* (Clay et al., 2016) (see Methods), to investigate further the transcriptomic signal established at basal expression contrasting the invasive genotypes from native genotypes biased towards biotic stress responses (Figures 1c, 5a and 6). We compared total 24 RNA‐seq profiles (i.e., two tissue types, two treatments, and six genotypes) in eight replicates (192 samples) generated for leaf and rhizome tissue separately harvested at preinoculation (basal expression) and post‐inoculation (response to biotic stress) to deduce DEGs and their representative enriched functions in six individual genotypes (see Methods, Supporting Information data set 4). In general, more DEGs were significantly induced (Figure 6a) than repressed (Figure 6b) in response to the endophyte inoculation. The two invasive genotypes IOH1 and IMI1 showed more attenuated responses to endophyte inoculation compared to the three native genotypes. However, the invasive genotype IMI3 showed a response similar in magnitude to the native genotype NMI2 (Figure 6a,b and Supporting Information data set 6). Further, IMI3 and the three native genotypes shared a substantial number of endophyte‐induced DEGs (Figure 6a red boxes).

We searched for enriched functional associations between endophyte‐induced and repressed genes in the invasive and native genotypes (Figure 6c and Supporting Information data set 6). In the native genotypes and IMI3 genotype, endophyte‐induced genes were enriched in “ATP binding” and “defence response”, while endophyte‐repressed genes were enriched in processes associated with photosynthesis (Figure 6c). A closer inspection into these induced genes annotated under GO molecular function “ATP‐binding” and GO biological process “defence response” revealed that many encode membrane receptor kinases known for roles in defence signalling (Supporting Information data set 6).

Despite genotypic variation in response to the endophyte inoculation, the native genotypes showed an overall stronger response in induced genes associated with biotic stress and defence, while these functions had higher basal expression in the invasive genotypes. Genotypes IMI3 and NMI2, while showing the highest number of induced DEGs annotated under defence response, also showed the highest number of repressed DEGs associated with photosynthesis compared to the rest of the genotypes, suggesting that the strong defence response concurrently downregulated photosynthesis in these two genotypes (Figure 6c–e). This is less prominent in NOH1 where both the number and enrichment of photosynthesis‐related endophyte‐repressed DEGs were among the smallest compared to other genotypes.

## 4 DISCUSSION

The genome of common reed (*Phragmites australis*) reported here provides the first reference genome for this ecologically important species, a genomic model for the model invasive grass *P*. *australis* spp. *australis* (Martin & Blossey, 2013; Saltonstall, 2002), and the first reference genome for the grass subfamily Arundinoideae (Figures 1 and 2, Table 1). Our genomic analyses, including nonreference allele frequency distributions and comparative genomics with other grasses, indicate that the reference genome was derived from a functionally diploid plant although *P*. *australis* has been generally reported as a tetraploid in North America (Clevering & Lissner, 1999). The reference genome provides a core set of reference genes within unique genomic backgrounds independent of chromosome‐level ploidy of contemporary polyploidy. However, it can be used as a fundamental genetic resource to tag unique genomic loci fluorescently and monitor chromosomal‐level variation, identify multivalent chromosomes, or study whether higher‐ploidy level populations can result from population‐specific additional successive WGD events, endoreplication found in clonal propagation, and/or introgression between populations.

Contemporary polyploids can show cytological diploidization (chromosomal changes leading to bivalent chromosome pairing as a cellular mechanism) or genic diploidization (a WGD event often followed by gene loss as a molecular mechanism), and these two mechanisms are independent from each other (Li et al., 2021; Ma & Gustafson, 2005; Tayalé & Parisod, 2013). The random shotgun sequencing of genomic DNA performed using Illumina reads suggest that the source DNA of the *P*. *australis* reference genome is derived from a functional diploid organism (Figure S1). Functional diploidy together with disomic inheritance is common in sexually reproducing plants even when they represent true polyploids, as shown for nearly half of polyploid species demonstrating bivalent chromosome pairing (Li et al., 2021). The status of *P*. *australis* as a functional diploid is in line with previous studies reporting cytological evidence of bivalent pairing of *P*. *australis* chromosomes between metaphase I and II during pollen development (Gorenflot, 1976) and karyotyping of cells from root tissues (Raicu et al., 1972).

WGD events are ubiquitous in land plant evolution at large, but less common within lineages, and mark important divergence points in lineage evolution while serving as a significant source for novel adaptations (Soltis & Soltis, 2016). We found a previously unreported whole genome duplication (WGD) event leading to the *P*. *australis* lineage, independent of the three ancient WGD events known in the grasses (McKain et al., 2016). The WGD event detected in the *P*. *australis* genome predates the divergence between spp. *australis* and *americanus* but postdates the divergence from the Panicoideae (Figures 3 and 4). As expected with substantial gene fractionation following WGD events, the *P*. *australis* genome lost up to 48% of its duplicated genes (Figure S2a) but retained over 14,005 duplicated genes. The high level of gene fractionation observed in the current reference genome suggests that it is a mesopolyploid (Li et al., 2021), given that *Phragmites* has undergone a more recent WGD that is still in the process of genic diploidization (Wang et al., 2011). The retained duplicated genes in the reference genome were strongly enriched with transcription factors and other genes associated with regulatory processes. Genes encoding transcription factors have been preferentially retained over other gene groups following paleo WGD events in other pan‐global species, such as *Arabidopsis thaliana* (Blanc & Wolfe, 2004; De Bodt et al., 2005; Freeling et al., 2007), and are thought to be a hallmark feature of WGD events underlying rapid diversification and global distribution of angiosperms (Birchler, 2019; Cheng et al., 2018). They may also provide a selective advantage in invasive species when introduced to new environments (Moura et al., 2021; te Beest et al., 2012). Therefore, the duplicated gene space detected in the *Phragmites* lineage enriched in regulatory genes provides novel genetic material for selection to act facilitating its potential as an invasive species.

Adaptive innovations initiated at the genomic level and further diversified at the transcriptome level can lead to distinct ecological fates. Our comparative analysis using native and invasive genotypes from the Great Lakes region of North America indicates that gene expression associated with defence against biotic stress is primed in the invasive genotypes compared to native genotypes. When a biotic stress response was induced by inoculation with the fungal endophyte *A. alternata*, the native genotypes were consistently more responsive (Figures 5 and 6). Altered interactions with pathogens and native plant species by invasive *Phragmites* could contribute to its success (Mangla & Callaway, 2008; Schroeder et al., 2020). A primed transcriptome with elevated transcript abundance for stress‐responsive genes has been recognized as an adaptive strategy exhibited by plants adapted to various abiotic or biotic stresses (Baccelli et al., 2020; Dräger et al., 2004; Wang, DiTusa, et al., 2021). A primed transcriptome for biotic stresses allows faster defence responses to an array of biotic stresses compared to an induced response from those species/populations that are not primed for these biotic stresses. Therefore, populations with primed transcriptomes to biotic stresses are expected to have a selective advantage over nonprimed populations if introduced to environments with a high biotic stress. It will be important to determine if the expression differences reported here are geographically variable and extend beyond the specific samples used in this study.

*Phragmites australis* is known for its intraspecific cytological ploidy polymorphism (Clevering & Lissner, 1999; Lambertini et al., 2006) coincident with its widespread success as a globally recognized invasive species. A higher gene content present in polyploids may predispose a species for invasive lifestyles adapted to a broad range of habitats potentially defined by both biotic and abiotic stresses (te Beest et al., 2012). However, whether ploidy level plays a deterministic role in facilitating invasiveness in *P*. *australis* remains debatable when categorical higher ploidy levels exclusively assigned to invasive genotypes are absent. For example, Gulf Coast *P*. *australis* subsp. *berlandieri* is cytologically hexaploid yet is not considered invasive, and cytologically octoploid *P*. *australis* has been documented along the Charles River in Massachusetts (Keller, 2000). A recent study (Wang, Wang, et al., 2021) showed that there were gene expression‐based functional enrichment differences related to environmental stress tolerance between tetraploid and octoploid *P*. *australis* populations. However, our comparative transcriptomics analysis does not limit the identification of traits associated with enriched transcripts in certain genotypes that may additionally have distinct ploidy levels because expression of genes is determined based on a unique genomic locus in the reference genome. This approach does not differ between transcriptomes analysed from diploid or polyploid organisms when the reference genome is created as a “haploid reference” assigned to the species. Further, the transcript expression observed from bulk tissues in plants generally include a mixture of cells at different ploidy levels regardless of the species ploidy level (De Rocher et al., 1990). Even diploid plants such as *Arabidopsis thaliana* tend to have higher ploidy cells exceeding the diploid cell fraction with developmental age (Galbraith et al., 1991). Expression differences related to tissue‐level polyploidy with plant age or induced by environmental stress, as well as species‐level polyploidy, also provide potential mechanisms for invasive species adaptation in new environments (De Rocher et al., 1990; Kettenring & Mock, 2012; te Beest et al., 2012). Exploring *P*. *australis* pangenomes in future studies could provide further insight into how genic or cytological ploidy polymorphism generates selective advantages for invasive genotypes of *P. australis* relative to noninvasive genotypes.

Our results provide a foundation for the development of novel species‐ and subspecies‐specific genetic approaches for control of invasive *Phragmites* (Harvey‐Samuel et al., 2017) and potentially other invasive plant species. There is widespread interest in controlling invasive *Phragmites* in many parts of its invasive range. Our genomic data help to identify unique and essential genes that could be targeted in genetic control approaches using RNAi with higher species specificity than attainable using chemical or mechanical control. Our transcriptomic data also point to potential target genes highly expressed in the invasive versus native genotypes. However, our results also provide some caution in particular approaches if targeted genes are duplicated or shared among both native and invasive groups. While RNAi approaches have been widely explored for controlling invasive insect pests and plant pathogens (Cagliari et al., 2019; Mamta & Rajam, 2017), to our knowledge, no RNAi‐based treatments have been developed to control problematic plants given the dearth of genomic data from invasive plant species. Future attempts at genetic control for invasive *Phragmites* should take into account that genetic variation exists in invasive *Phragmites* genotypes and that native genotypes can co‐occur in the same habitats.

## CONFLICT OF INTEREST

The authors declare no competing interests. Any use of trade, product, or firm names is for descriptive purposes only and does not imply endorsement by the U.S. Government.

## AUTHOR CONTRIBUTION

Keith Clay, Maheshi Dassanayake and Kurt P. Kowalski conceived and designed the experiments and obtained the funding. Philippa Tanford., Keith Clay, and Kurt P. Kowalski conducted data collections and related experiments. Dong‐Ha Oh, Chathura Wijesinghege, and Maheshi Dassanayake designed the bioinformatic work flows and performed computational analyses. Dong‐Ha Oh and Quynh N. Quach generated figures. All authors contributed to drafting and finalizing the manuscript.

### OPEN RESEARCH BADGES

This article has earned an Open Data Badge for making publicly available the digitally‐shareable data necessary to reproduce the reported results. The data is available at https://doi.org/10.6084/m9.figshare.14036756, https://www.ncbi.nlm.nih.gov/bioproject/?term=PRJNA705976 and https://genomevolution.org/coge/GenomeInfo.pl?gid=59768.

## Supporting information

Supplementary Material

## Data Availability

All raw and assembled sequence data have been deposited to NCBI under BioProject PRJNA705976. In addition, the reference genome sequence, gene models, and representative RNA‐Seq tracks are available in the CoGe database (https://genomevolution.org/coge/) with the Genome ID 59768, for browsing and other CoGe‐embedded comparative analyses (Nelson et al., 2018). Large Supporting Information data sets have been deposited in Figshare (https://doi.org/10.6084/m9.figshare.14036756) and also in the USGS ScienceBase repository (https://doi.org/10.5066/P9NLU6Q4).
