# Supplementary material for: Novel genome characteristics contribute to the invasiveness of Phragmites australis (common reed)
Source: Mol Ecol. 2021 Dec 11;31(4):1142–59. doi: 10.1111/mec.16293 (PMC9300010; doi:10.1111/mec.16293)
Supplement: Supplementary file 1 — Supplementary Material [file MEC-31-1142-s001.pdf]

Supplementary Materials for: Novel genome characteristics contribute to the invasiveness of *Phragmites australis* (common reed)

Dong-Ha Oh, Kurt P. Kowalski, Quynh N. Quach, Chathura Wijesinghege, Philippa Tanford, Maheshi Dassanayake, and Keith Clay

Supplementary Methods

Propagation of *Phragmites australis* genotypes in growth chambers

Phragmites invasive and native genotypes were propagated from rhizome cuttings at Indiana University and maintained in large clay pots in the Indiana University Greenhouse. Rhizome cuttings with developing buds (3-10cm in length) were planted in potting soil in round plastic pots (9cm height, 11cm diameter) and watered as needed. Potted cuttings were kept in the greenhouse until transfer to a Percival Scientific Model WE-1012 Environmental Controller walk-in growth chamber kept at 70°F and 70% relative humidity setting with a 14:10 hour light:dark cycle for approximately 60 days before further manipulations.

Endophyte treatment and RNA sample preparation

Endophyte treatments. Replicate plants from all genotypes were inoculated with an *Alternaria alternata* fungal endophyte (Accession KT923239), one of the most abundant fungal OTUs isolated from *Phragmites* (Clay et al., 2016), to potentially elicit *Phragmites* transcriptional responses. Fungal inoculum from stock cultures was used to inoculate four flasks with 50mL liquid culture medium (M102) (Bacon, 2018) in 1L distilled H_2O, which were then cultured for 4 days on a Braun Melsungen AG auto shakeR Type 886013 shaker table at 120 rpm. One tiller per plant was inoculated using three methods. First, a sterile 1ML needle-tipped syringe covered in *Alternaria alternata* mycelium was inserted into the tiller above the first node. Second, a sterile wooden toothpick overgrown with mycelium from liquid culture was inserted above the second node and left in place overnight. Finally, three sequential leaves on the inoculated tiller were rubbed with sterile chromatography sand to create small wounds to the leaf epidermis, and the entire plant was treated with inoculum spray. Spray inoculum was prepared by decanting the contents of four 50mL liquid culture flasks into 50 mL centrifuge tubes (~30mL/tube). Sterile DI water was added to each tube and the contents were thoroughly mixed

by shaking, centrifuged, and the supernatant discarded. This process was repeated a second time and 500mL DI water was added to the rinsed mycelium and blended for 30 seconds in a sterile blender, and transferred to a 550mL plastic spray bottle for several hours before application. Abundant mycelium was visible on a microscope slide at 200X magnification following a single spray, but few or no fungal spores were present. Control plants received identical treatments but with sterile needles, sterile toothpicks, and a 2% liquid culture media solution in sterile DI water. Treated plants were then covered and secured in a plastic bag overnight to maintain high humidity. The growth chamber was kept dark for 36 hours after inoculation before resuming a 14:10 hour light/dark cycle.

Plant tissue sampling and inoculum isolation. Two inoculated and two control plants per genotype were sampled eight days after inoculation treatment applications for transcriptome analysis. Soil was washed away in tap water and the plant was blotted dry before the treated tillers and attached rhizome were processed. We sampled the first 2cm of rhizome directly connected to the infected tiller. If more rhizome tissue was present, 0.5cm rhizome sections were taken every 2cm or until a total of 5cm was sampled (2cm + six 0.5cm samples). The rhizome sections were cut in half lengthwise with a clean razor blade to replicate samples for RNA extraction. Three leaves from the infected tiller were each cut into quarters after a sample was taken to confirm inoculation. The top and bottom quarter of each leaf were pooled to replicate samples for RNA extraction.

Leaf tissue samples were taken from the three inoculated leaves per plant rubbed with sand to confirm the effectiveness of inoculation. A 1cm^2 leaf section from each leaf was cut into small fragments, placed in small plastic tissue cassettes and rinsed for 20 minutes under running tap water individually. Leaf fragments within the tissue cassettes were then washed for 1 minute in 95% ethanol, 3 minutes in 0.18% sodium hypochlorite, 1 minute in 95% ethanol, and 2 minutes in sterile DI water following Christian et al. (2019). Each leaf fragment was removed from the tissue cassette, air-dried on a sterile paper towel and placed on the surface of a corn meal agar (CMA) petri plate supplemented with 25mg tetracycline/L for 5 minutes to confirm the efficacy of surface sterilization. Each fragment was then cut in half with a sterile razor to expose

the plant interior and plated on another CMA + tetracycline plate to determine if subsequent fungal growth corresponds to the original *Alternaria alternata* inoculum.

RNA Extraction. Tissue samples were grounded in liquid nitrogen in porcelain mortars and pestles while the mortar was resting in dry ice. Mortars and pestles were previously wrapped in aluminum foil and baked in a drying oven at 180°C for six hours to destroy RNase and then pre-chilled in a -20°C freezer. Grounded tissue and liquid nitrogen was decanted into a 2mL microcentrifuge tube and liquid nitrogen was allowed to evaporate. ~1mL of RLT buffer with 10µl 2-mercaptoethanol per mL was then added. Each sample was promptly vortexed and put on ice until transfer to long-term storage at -80°C. Between samples (except replicates), mortars and pestles were washed with soap and water, sprayed with RNase AWAY (Molecular BioProducts, Inc.), and re-chilled. Most samples were processed within ten minutes of cutting.

RNA was extracted using the RNeasy Plant Mini Kit (Qiagen) following the manufacturer's protocol with the following minor modifications. Samples were thawed on ice, and centrifuged for 15-30 seconds at 10,000xg to pellet plant tissue and allow for pipetting of the supernatant. Then, 300 µl of sample was added to the Qiashredder column (up to ~800ul for samples with lower concentration). Extracts were stored at -80°C in RNase-free tubes.

RNA Library preparation and sequencing. cDNA libraries were prepared with the Illumina Stranded mRNA Prep Kit (Illumina) following the standard protocol with half volume reactions and 12 PCR amplification cycles in the library enrichment step. Libraries from samples of low concentration (<500ng material available) were constructed from 25ul of input material regardless of concentration. Final library quality and concentration for pooling were assessed with Agilent D1000 ScreenTape (Agilent). Pooled libraries were sequenced through a NextSeq 75 cycle high-output run with paired-end read on an Illumina NextSeq500 at the Center for Genomics and Bioinformatics at Indiana University.

<u>Reference genome assembly</u>

For the reference genome sequencing and assembly, we chose the PacBio sequencing platform to benefit from its long read lengths. We selected Canu as the primary genome assembler so that the error-prone PacBio long reads can be corrected based on the consensus

among themselves (Koren et al., 2017). To ensure efficient correction in the hierarchical assembly by Canu without the need for a secondary sequencing technology, we produced PacBio reads that represent a more than 40-fold sequencing depth for a haploid genome size 1Gb, estimated based on the 1C value of 1.01pg reported for *Phragmites australis* (Pyšek et al., 2018). The completeness of the resulting primary assembly was confirmed by the high complete BUSCO content (93.3%; Fig. 3 and Supplementary Table 1) and RNA-seq read mapping rates (~89%; Supplementary Table 5).

We report all assembled contigs as the reference genome sequences without an additional purging step. While there has been a concern recently raised on incomplete haplotig and duplicate purging for genome assemblies based on long reads (Guiglielmoni et al., 2021; Murigneux et al., 2020), currently available tools for haplotig/duplicate purging have not been designed nor tested for highly repetitive plant genomes with either polyploidy or recent whole-genome duplication and so could result in over-purging true duplicated genomic regions (Guan et al., 2020; Roach et al., 2018).

Determination of non-reference allele frequencies in single nucleotide polymorphism (SNP) loci.

Before gene model prediction, we aligned paired-end short reads (150 nucleotides x2) derived from the same genomic DNA samples used for the reference genome assembly, to the reference genome sequence using bowtie2 (v. 2.2.6) (Langmead & Salzberg, 2012). We then determined non-reference allele frequencies in genomic regions with homologies to Benchmarking Universal Single-Copy Ortholog (BUSCO) sequences (Chan et al., 2017) using mpileup command of samtools (v. 1.8) with a Phred quality score cutoff of 20 (Li, 2011) (Supplementary Fig. 1). To determine the genomic regions harboring putative BUSCOs, we first detected transcripts homologous to the 956 BUSCO HMM profiles (Chan et al., 2017) from the "Invasive combined" transcriptome (Supplementary Table 2). These transcripts were mapped to the genomic regions using GMAP (v. 2012-04-21) (Wu & Watanabe, 2005).

Identification of "Conserved" and "Duplicate" groups among *P. australis* reference gene models.

Using OrthoFinder (v. 2.2.7) (Emms & Kelly, 2019) and MMseqs2 (Steinegger & Söding, 2017), we identified ortholog groups (OGs) among deduced protein sequences from the primary

reference gene models of *P. australis* and five comparator monocot species. For each OG, we calculated the ratio of ortholog copy number (R_{CN}) for each species, as defined as the ortholog copy number in a species divided by the mean ortholog copy number of all other species (Fig. 4a). OGs with the standard deviation of ortholog copy number across all species exceeding 10% of the total number of orthologs were excluded since such OGs tend to include lineage-specific expansion of transposable element-like genes whose copy numbers are less relevant with the *P. australis*-specific whole genome duplication (WGD) event (Fig. 3 and Supplementary Dataset 2). Among *P. australis* OGs, we identified "Conserved" and "Duplicated" groups whose R_{CN} values are between 0.75 to 1.25 ("Conserved") and larger than 1.5 ("Duplicated"), respectively (Fig. 4a and Supplementary Table 1), representing *P. australis* orthologs whose copy numbers have been fractionated to the level similar to other monocot species ("Conserved") or retained as duplicated after the WGD event.

References

Bacon, C. W. (2018). *Biotechnology of Endophytic Fungi of Grasses*. CRC Press. https://play.google.com/store/books/details?id=4EUPEAAAQBAJ

Chan, K.-L., Rosli, R., Tatarinova, T. V., Hogan, M., Firdaus-Raih, M., & Low, E.-T. L. (2017). Seqping: gene prediction pipeline for plant genomes using self-training gene models and transcriptomic data. *BMC Bioinformatics*, *18*(1), 1426. https://doi.org/10.1186/s12859-016-1426-6

Christian, N., Herre, E. A., & Clay, K. (2019). Foliar endophytic fungi alter patterns of nitrogen uptake and distribution in *Theobroma cacao*. *The New Phytologist*, *222*(3), 1573–1583. https://doi.org/10.1111/nph.15693

Clay, K., Shearin, Z. R. C., Bourke, K. A., Bickford, W. A., & Kowalski, K. P. (2016). Diversity of fungal endophytes in non-native *Phragmites australis* in the Great Lakes. *Biological Invasions*, *18*(9), 2703–2716. https://doi.org/10.1007/s10530-016-1137-y

Emms, D. M., & Kelly, S. (2019). OrthoFinder: phylogenetic orthology inference for comparative genomics. *Genome Biology*, *20*(1), 238. https://doi.org/10.1186/s13059-019-1832-y

Guan, D., McCarthy, S. A., Wood, J., Howe, K., Wang, Y., & Durbin, R. (2020). Identifying and removing haplotypic duplication in primary genome assemblies. *Bioinformatics*, *36*(9), 2896–2898. https://doi.org/10.1093/bioinformatics/btaa025

Guiglielmoni, N., Houtain, A., Derzelle, A., Van Doninck, K., & Flot, J.-F. (2021). Overcoming uncollapsed haplotypes in long-read assemblies of non-model organisms. *BMC Bioinformatics*, *22*(1), 303. https://doi.org/10.1186/s12859-021-04118-3

Koren, S., Walenz, B. P., Berlin, K., Miller, J. R., Bergman, N. H., & Phillippy, A. M. (2017). Canu: scalable and accurate long-read assembly via adaptive k-mer weighting and repeat

separation. *Genome Research*, *27*(5), 722–736. https://doi.org/10.1101/gr.215087.116
Langmead, B., & Salzberg, S. L. (2012). Fast gapped-read alignment with Bowtie 2. *Nature Methods*, *9*(4), 357–359. https://doi.org/10.1038/nmeth.1923
Li, H. (2011). A statistical framework for SNP calling, mutation discovery, association mapping and population genetical parameter estimation from sequencing data. *Bioinformatics* , *27*(21), 2987–2993. https://doi.org/10.1093/bioinformatics/btr509
Love, M. I., Huber, W., & Anders, S. (2014). Moderated estimation of fold change and dispersion for RNA-seq data with DESeq2. *Genome Biology*, *15*(12), 550. https://doi.org/10.1186/s13059-014-0550-8
Maere, S., Heymans, K., & Kuiper, M. (2005). BiNGO: a Cytoscape plugin to assess overrepresentation of gene ontology categories in biological networks. Bioinformatics, 21(16), 3448–3449. https://doi.org/10.1093/bioinformatics/bti551
Murigneux, V., Rai, S. K., Furtado, A., Bruxner, T. J. C., Tian, W., Harliwong, I., Wei, H., Yang, B., Ye, Q., Anderson, E., Mao, Q., Drmanac, R., Wang, O., Peters, B. A., Xu, M., Wu, P., Topp, B., Coin, L. J. M., & Henry, R. J. (2020). Comparison of long-read methods for sequencing and assembly of a plant genome. *GigaScience*, *9*(12). https://doi.org/10.1093/gigascience/giaa146
Pyšek, P., Skálová, H., Čuda, J., Guo, W.-Y., Suda, J., Doležal, J., Kauzál, O., Lambertini, C., Lučanová, M., Mandáková, T., Moravcová, L., Pyšková, K., Brix, H., & Meyerson, L. A. (2018). Small genome separates native and invasive populations in an ecologically important cosmopolitan grass. *Ecology*, *99*(1), 79–90. https://doi.org/10.1002/ecy.2068
Roach, M. J., Schmidt, S. A., & Borneman, A. R. (2018). Purge Haplotigs: allelic contig reassignment for third-gen diploid genome assemblies. *BMC Bioinformatics*, *19*(1), 460. https://doi.org/10.1186/s12859-018-2485-7
Steinegger, M., & Söding, J. (2017). MMseqs2 enables sensitive protein sequence searching for the analysis of massive data sets. *Nature Biotechnology*, *35*(11), 1026–1028. https://doi.org/10.1038/nbt.3988
Wu, T. D., & Watanabe, C. K. (2005). GMAP: a genomic mapping and alignment program for mRNA and EST sequences. *Bioinformatics*, *21*(9), 1859–1875. https://doi.org/10.1093/bioinformatics/bti310

Supplementary Table 1 | Monocot genomes for comparative analyses

Species	Version	Source	BUSCO (%) [b]	Protein-coding gene loci
Ananas comosus [a]	3	Phytozome (ID:321)	C:91.8[S:87.4,D:4.4],F:3.6,M:4.6	27,024
Brachypodium distachyon [a]	3.1	Phytozome (ID:314)	C:99.5[S:98.3,D:1.2],F:0.5,M:0.0	34,310
Cenchrus americanus	1.0	PLAZA (4.5)	C:82.3[S:79.8,D:2.5],F:11.3,M:6.4	38,579
Hordeum vulgare	2.44	IBSC	C:84.8[S:80.1,D:4.7],F:3.9,M:11.3	36,760
Leersia perrieri	N/A	Ensembl (r42)	C:92.5[S:90.8,D:1.7],F:3.7,M:3.8	29,176
Oryza sativa Japonica [a]	7	Phytozome (ID:323)	C:95.1[S:93.7,D:1.4],F:3.4,M:1.5	42,189
Oropetium thomaeum	1.0	Phytozome (ID:386)	C:81.2[S:78.4,D:2.8],F:13.7,M:5.1	28,437
Phragmites australis [a]	1.0	This study	C:93.3[S:56.6,D:36.7],F:4.9,M:1.8	64,857
Phyllostachys edulis	1.0	NCGR	C:73.5[S:61.1,D:12.4],F:13.7,M:12.8	31,987
Sorghum bicolor [a]	3.1.1	Phytozome (ID:454)	C:99.3[S:97.8,D:1.5],F:0.4,M:0.3	34,129
Setaria italica [a]	2.2	Phytozome (ID:312)	C:98.4[S:96.7,D:1.7],F:1.1,M:0.5	34,584
Saccharum spontaneum	v20190103	PLAZA (4.5)	C:92.4[S:22.7,D:69.7],F:3.4,M:4.2	83,826
Triticum aestivum	1.1.44	IWGSC	C:99.6[S:1.1,D:98.5],F:0.1,M:0.3	107,327
Zea mays B73	4	AGP	C:94.3[S:84.9,D:9.4],F:3.9,M:1.8	39,421
Zoysia japonica ssp. *nagirizaki*	1.1	PLAZA (4.5)	C:45.9[S:41.0,D:4.9],F:28.9,M:25.2	59,271

[a] Selected for comparative analyses in Fig. 3 and Fig. 4.

[b] Based on "embryophyta odb10" with total 1,375 Benchmarking Universal Single-Copy Orthologs (BUSCOs), shown are percentages of complete (C) single-copy (S), duplicated (D), fragmented (F), and missing (M) BUSCOs.

Supplementary Table 2 | *De novo* assembled *P. australis* transcriptomes

Transcriptome	# ORFs	min (nt)	median (nt)	mean (nt)	N50 (nt)	max (nt)	sum (nt)
Invasive reference [a]	97,076	123	678	895	1,230	14,250	86,956,037
Native leaf [b]	49,224	255	672	919	1,155	12,597	45,275,301
Native rhizome	56,916	258	768	998	1,263	13,053	56,843,553
IOH1 leaf [c]	34,131	255	699	950	1,191	12,600	32,448,375
IOH1 rhizome	27,866	258	744	1,009	1,290	8,745	28,125,045
IMI1 leaf	35,114	255	702	956	1,209	12,600	33,576,678
IMI1 rhizome	44,499	258	804	1,040	1,308	13,548	46,311,834
IMI3 leaf	34,890	258	717	964	1,212	13,053	33,665,640
IMI3 rhizome	39,165	261	810	1,031	1,281	12,648	40,404,453
NOH1 leaf	39,466	255	690	939	1,185	12,918	37,085,379
NOH1 rhizome	46,233	255	798	1,024	1,275	12,762	47,376,243
NMI1 leaf	42,354	261	708	963	1,218	12,795	40,798,848
NMI1 rhizome	48,766	261	789	1,030	1,305	13,053	50,252,232
NMI2 leaf	43,633	258	690	939	1,185	12,777	40,976,049
NMI2 rhizome	37,619	255	735	994	1,266	15,036	37,424,133

Stats for open reading frames (ORFs) for all *de novo* assembled transcriptomes.

[a] RNA-seq reads (150nt x2) from leaf, shoot, and rhizomes of the same clone used for genome assembly were assembled with Trinity (v. 2.4.0) and SPAdes (v. 3.10.0) and consolidated using the evigene pipeline. This transcriptome was used as the "EST hints" for reference gene model annotation. [b] Half of all RNA-seq reads (37nt x2) from the three native genotypes were combined and assembled as described in Methods. The Native rhizome transcriptome was the representative of *P. australis* native subspecies (ssp. *americanus*) in Fig. 3c and 3d. Using the Native leaf transcriptome produced similar results.

[b] All RNA-seq reads (37nt x2) from each tissue were assembled to transcriptomes for each genotype, as described in Methods. The rhizome transcriptomes represented the six genotypes in Fig 5b. The leaf transcriptomes produced a near identical tree.

Supplementary Table 3 | Proportion of transposable elements in the *Phragmites australis* reference genome

Category	Number of elements	Length occupied (nt)	Percent occupied (%)
SINEs	8,937	1,643,956	0.14
LINEs	33,384	19,841,853	1.74
LTR elements	326,358	415,123,549	36.42
DNA elements	299,783	130,261,850	11.43
Unclassified	214,476	73,623,178	6.46
Total interspersed repeats		640,494,386	56.19
Total sequence except gaps		1,139,927,050	100.00

Supplementary Table 4 | Comparison of orthologous gene copy numbers among *P. australis* and five monocot genomes

Copy number ratio compared to others (R_{CN})[a]	Number of Ortholog groups (OGs)					
	A.comosus	*B. distachyon*	*O. sativa*	*P. australis*	*S. bicolor*	*S. italica*
0.0≤ R_{CN} ≤0.125	1136	59	73	92	20	26
0.125< R_{CN} ≤0.25	44	3	2	0	1	1
0.25< R_{CN} ≤0.375	145	23	13	11	12	5
0.375< R_{CN} ≤0.5	892	248	194	103	133	80
0.5< R_{CN} ≤0.625	798	751	696	301	673	611
0.625< R_{CN} ≤0.75	573	823	800	259	809	741
0.75< R_{CN} ≤0.875	2600	3061	3052	851	3039	2985
0.875< R_{CN} ≤1.0	3134	3539	3568	3051	3613	3603
1.0< R_{CN} ≤1.125	139	395	433	278	461	495
1.125< R_{CN} ≤1.25	414	1562	1558	1463	1661	1680
1.25< R_{CN} ≤1.375	55	45	64	96	56	78
1.375< R_{CN} ≤1.5	284	272	295	475	290	325
1.5< R_{CN} ≤1.625	14	14	18	45	26	15
1.625< R_{CN} ≤1.75	430	181	205	943	184	260
1.75< R_{CN} ≤1.875	29	29	28	226	30	41
1.875< R_{CN} ≤2.0	371	109	106	2118	103	149
2.0< R_{CN} ≤2.125	2	3	2	18	2	5
2.125< R_{CN} ≤2.25	21	13	21	246	16	25
2.25< R_{CN} ≤2.375	0	4	2	33	4	5
2.375< R_{CN} ≤2.5	66	14	18	440	14	18
2.5< R_{CN} ≤2.625	0	0	0	0	0	0
2.625< R_{CN} ≤2.75	0	0	0	8	0	0
2.75< R_{CN} ≤2.875	1	0	0	91	1	0
2.875< R_{CN}	0	0	0	0	0	0

[a] Orthologous gene copy number (R_{CN}) was calculated for each species in an OG, by dividing the ortholog in the species by the mean copy number in all other species. The number of OGs in different R_{CN} ranges is shown for all species. For *P. australis*, OGs in "Conserved" and "Duplicated" groups are shaded in orange and skyblue, respectively. See Supplementary Methods for details.

Supplementary Table 5 | Alignments of RNA-seq reads derived from invasive and native genotypes to the *P. australis* reference genome and protein-coding gene model sequences

ID	Genotype	Total million read pairs (mean ± s.d.) [a]	% overall alignment rate [b] (mean ± s.d.)		
			vs. genome	vs. transcript model	vs. ORF model
IOH1	Invasive	29.55 ± 4.34	89.82 ± 3.44	73.65 ± 1.87	65.31 ± 2.06
IMI1	Invasive	28.48 ± 3.26	89.52 ± 3.02	72.43 ± 1.70	63.96 ± 1.89
IMI3	Invasive	28.88 ± 2.82	87.43 ± 5.36	71.95 ± 4.13	63.64 ± 3.83
NOH	Native	29.05 ± 3.92	78.32 ± 8.72	68.72 ± 7.75	61.07 ± 6.95
NMI1	Native	28.36 ± 3.17	80.65 ± 1.21	70.87 ± 1.52	63.24 ± 1.35
NMI2	Native	30.84 ± 6.56	81.21 ± 1.26	70.82 ± 1.20	63.21 ± 1.47

[a] Mean and standard deviation (s.d.) for total 32 RNA-seq samples per genotype, i.e. two tissues (leaf and rhizome) x two treatment (control and inoculated with endophytes) x eight replicates;

[b] RNA-seq reads were aligned to the reference genome, transcript model, or protein-coding open reading frame (ORF) model sequences. We used hisat2 for alignment to the genome sequence, while alignment to transcript and ORF model sequences was performed with bowtie2 ("--very-sensitive" option), as detailed in the Methods.

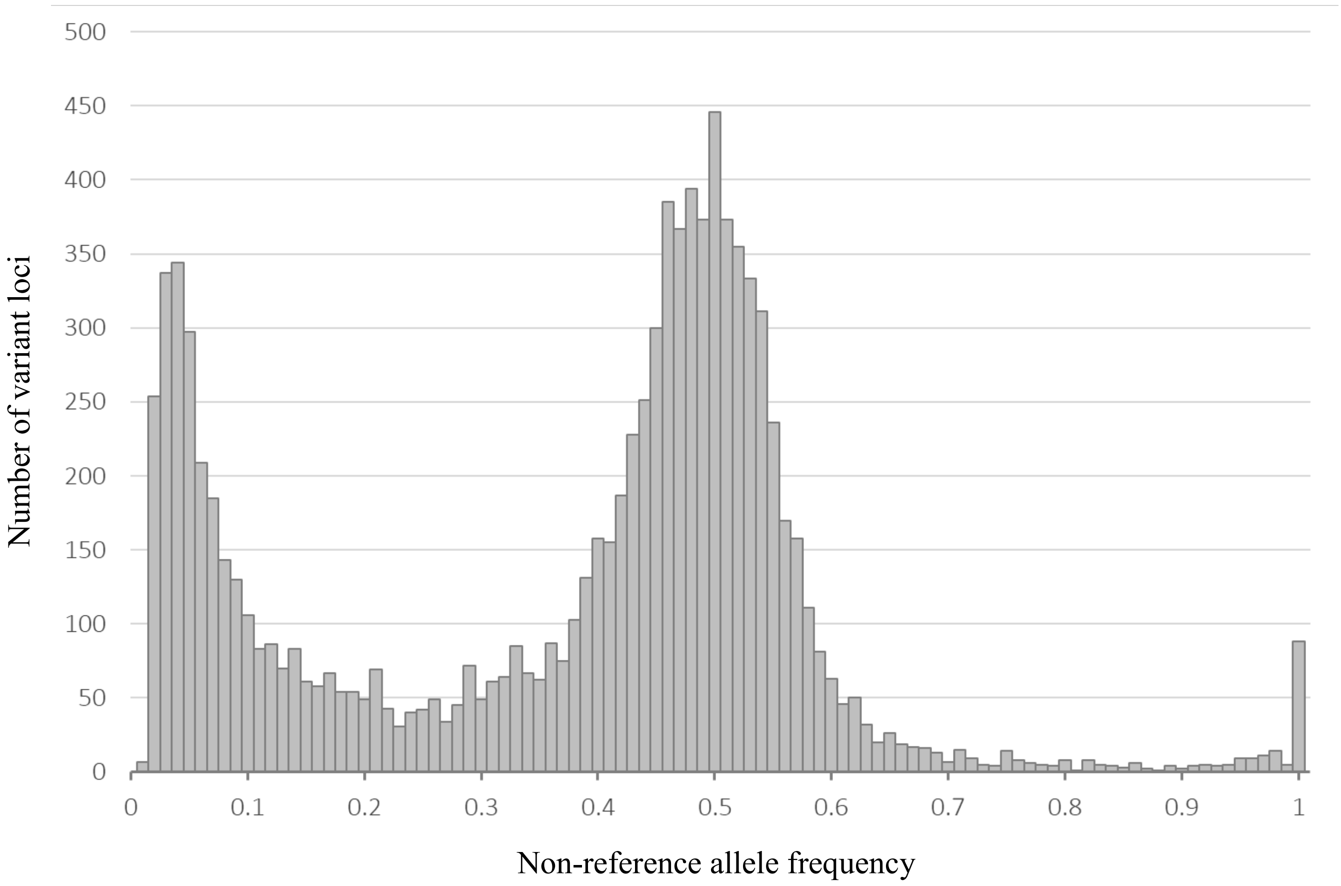

Supplementary Fig. 1 | Distribution of non-reference allele frequencies indicates a functionally diploid genome of the *P. australis* genotype used for the reference genome assembly. We identified total 9,835 single nucleotide polymorphisms in total 3,780,373-bp genomic regions harboring similarities with 699 BUSCOs and plotted the distribution of non-reference allele frequencies (see Supplementary Methods for details). The single major peak at allele frequency 0.5 coincided with the expectation from a functionally diploid genome.

a

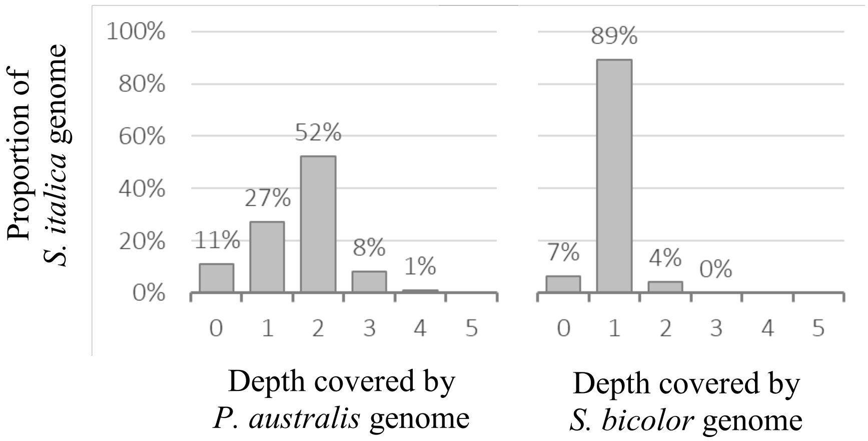

b

Contig size (# protein-coding gene loci)	Number of contigs	Number (%) of contigs with a synteny block
≥5	3551	1357 (38.2)
≥10	1549	1035 (66.8)
≥20	590	541 (91.7)
≥50	107	106 (99.1)
≥100	22	22 (100)

c

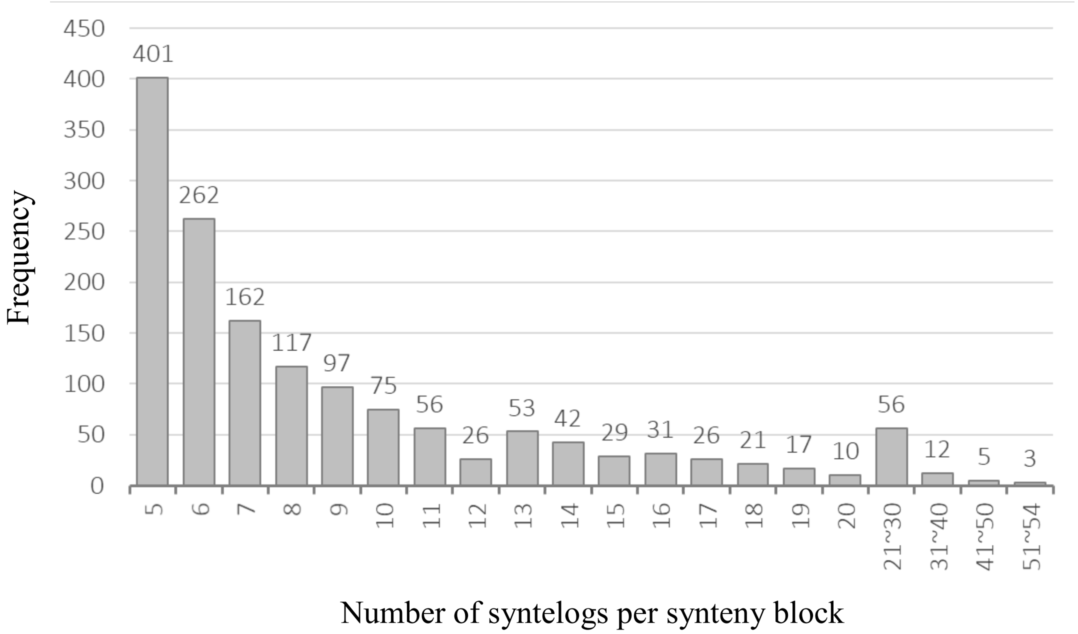

Supplementary Fig. 2 | Properties of synteny blocks identified in the *P. australis* genome. a, Comparison of the *Setaria italica* genome proportions covered by different depths with *Phragmites australis* and *Sorguhm bicolor* genomes, respectively. Total 52% of *S. italica* genes

were paired with two *P. australis* co-linear orthologs as exemplified in Fig. 3b, while most (>89%) of *S. italica* genes were 1:1 matched with a *S. bicolor* co-linear ortholog. **b,** Number and proportion of *P. australis* draft genome contigs that include a synteny block with minimum five co-linear paralogs. **c,** Size distribution of synteny blocks. Synteny blocks, detected using SynMap, are defined as a stretch of minimum five co-linear paralogs (i.e. syntelog) in other parts of the same genome. See the CoGe SynMap description (https://genomevolution.org/wiki/index.php/SynMap) for details.

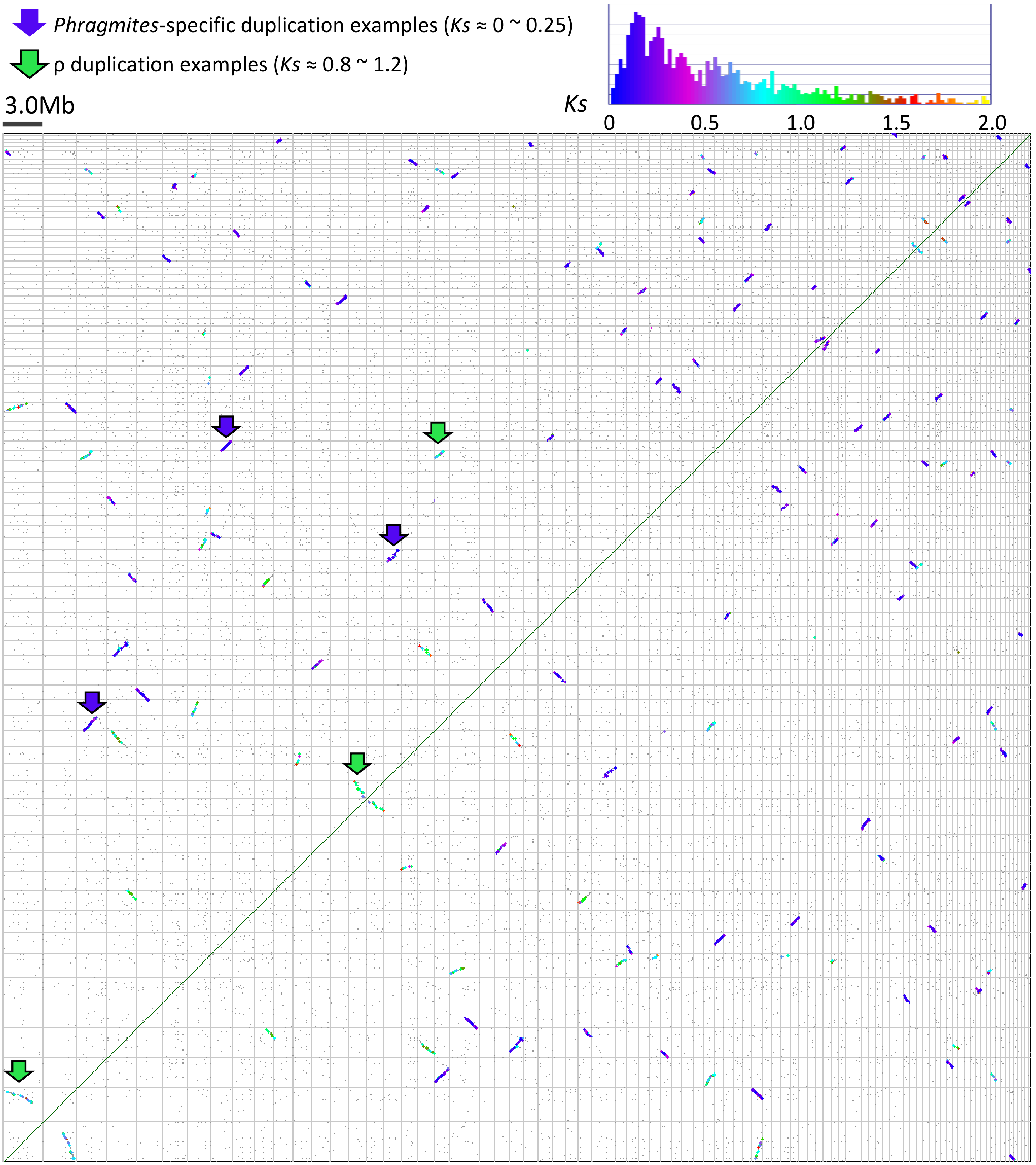

Phragmites-specific duplication examples (Ks ≈ 0 ~ 0.25)
ρ duplication examples (Ks ≈ 0.8 ~ 1.2)
3.0Mb
Ks
0
0.5
1.0
1.5
2.0

Supplementary Fig. 3 | The fifty largest synteny blocks detected within the *P. australis* genome. *P. australis* genome contigs harboring the fifty longest synteny blocks were compared to themselves using SynMap (https://genomevolution.org/coge/SynMap.pl) and visualized as a dot plot with each syntelog pair shown a dot color-coded based on the synonymous substitution rates (*Ks*) between the pair. The histogram (upper panel) shows both the color keys and distribution for *Ks*. Example synteny blocks representing *P. australis*-specific duplication and the ρ duplication shared among the grass lineage are marked with arrows.

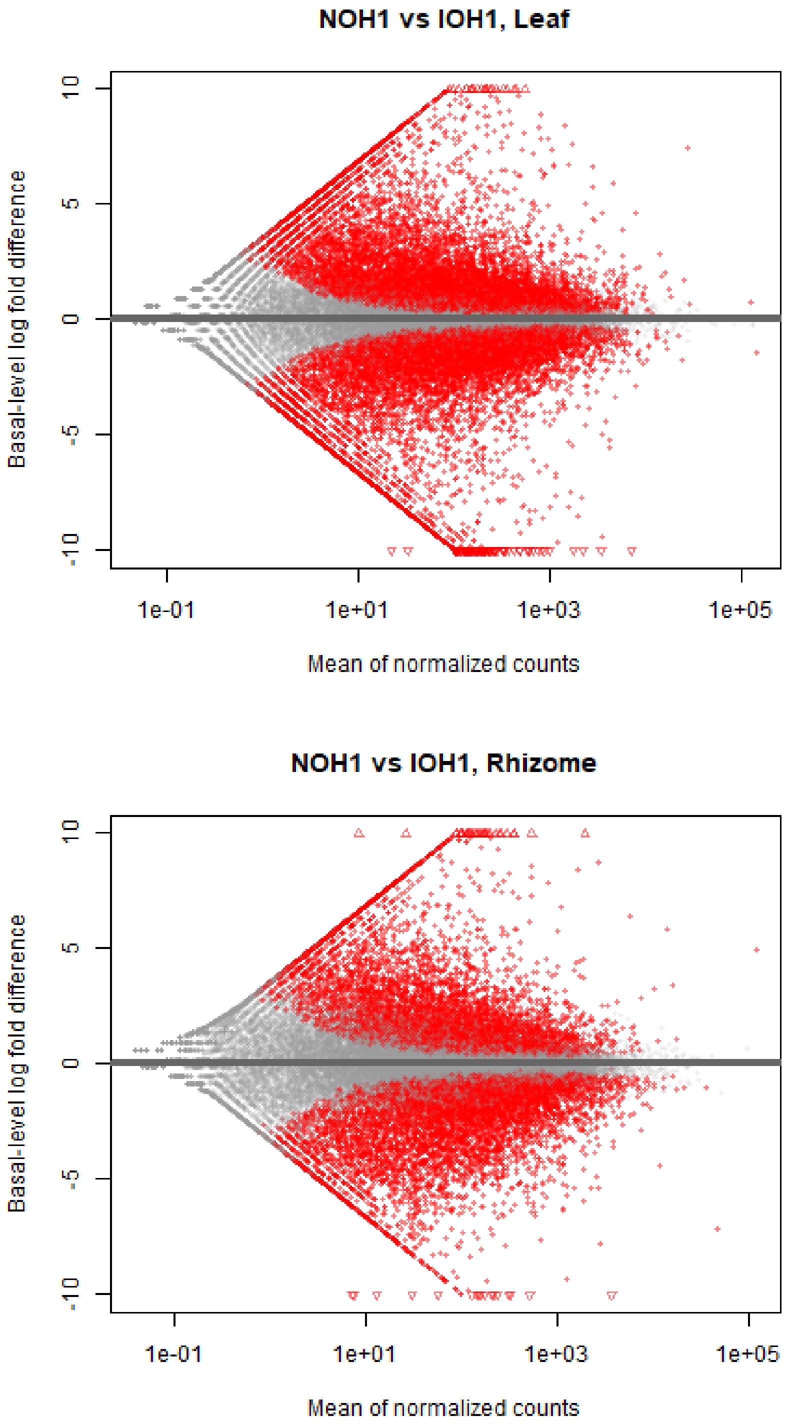

Supplementary Fig. 4 | RNA-seq analysis comparing basal-level expression among native and invasive *P. australis* genotypes. Representative "MA"-plots (Love et al., 2014) showing distribution of $\log_2$ fold differences (y-axis) and mean normalized RNA-seq counts (x-axis) for NOH1 and IOH1 genotypes. RNA-seq reads from both NOH1 and IOH1 genotypes were aligned

to the reference genome sequence and basal-level expression between genotypes were compared after normalization using total number of aligned reads, as detailed in Methods. Reference genes with significantly different basal-level expressions (adjusted p-value<0.05, estimated by DESeq2 (Love et al., 2014)) are in red. Note that despite the relatively lower percentage of total reads aligned to the reference genome sequences among native genotypes compared to invasive genotypes (Supplementary Table 2), the MA-plots did not show notable shifts towards lower basal-level expression in the native genotype. All other native and invasive genotype pairs showed similar patterns, corroborating the similar numbers of significantly differentially expressed genes (DEGs) between native and invasive genotype pairs in Fig. 5d.

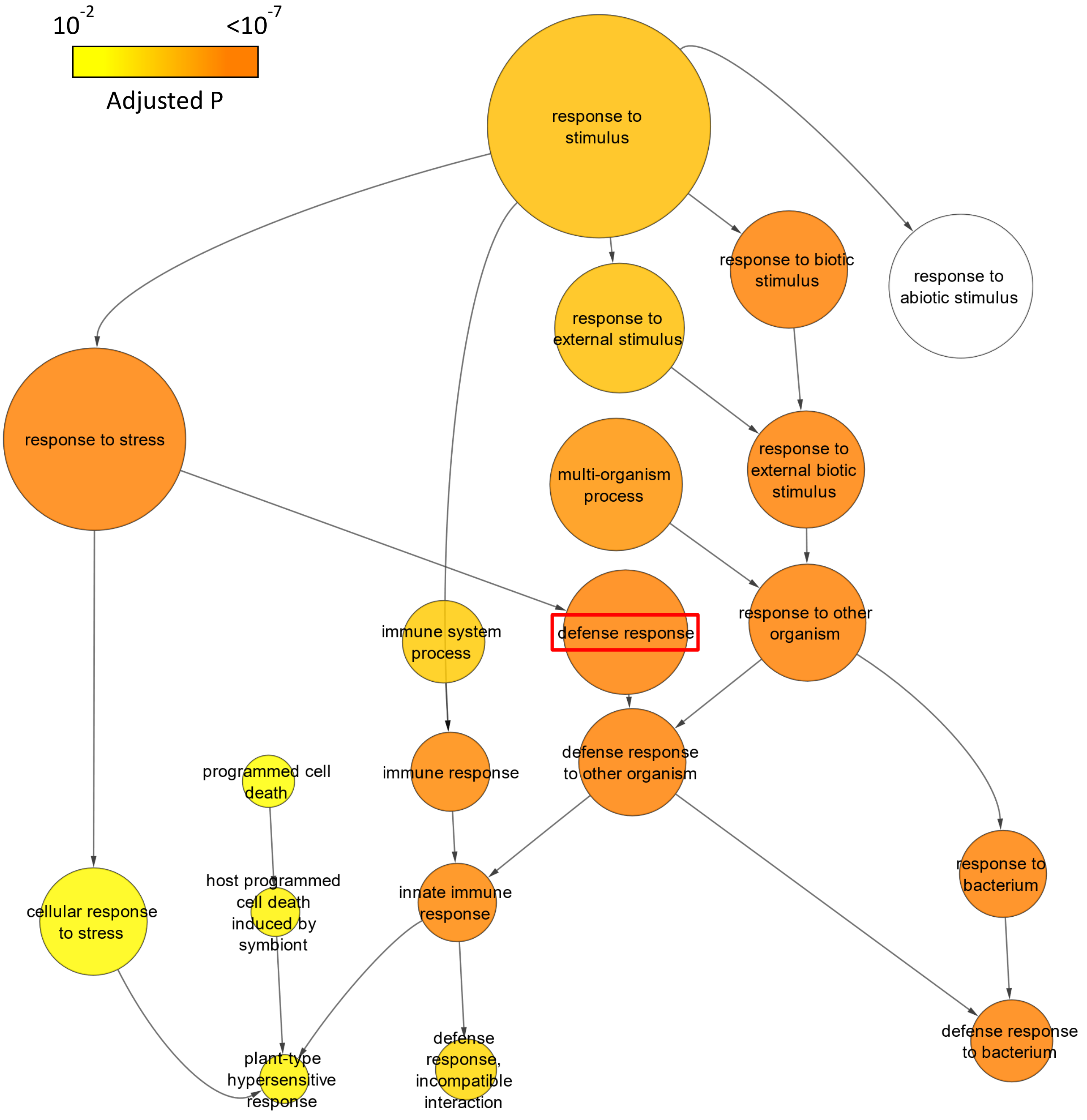

Supplementary Fig. 5 | An example network of Gene Ontology (GO) terms enriched among *P. australis* reference genes with higher basal-level expression in an invasive genotype. The network generated using BiNGO (Maere et al., 2005) where non-white colors indicate sighnificant adjusted p-value of enrichment with among *P. australis* reference genes with higher basal-level expression in the leaf samples of the IOH1 than the NOH1 genotype. Each GO term was indicated as a circle node, with its diameter mirroring the size of the GO term, and connected

to its parent and child GO terms. Note that among the child GO terms of “response to stimulus,” defense-related GO terms, e.g. “defense response” (red box), showed the highest levels of enrichment, while other child terms such as “response to abiotic stimulus” was not significantly enriched. For number of genes in each GO terms and detailed p-values for genes showing different basal-level expression between all genotype pairs, see Supplementary Dataset 4.

The following datasets are available at Figshare (https://doi.org/10.6084/m9.figshare.14036756) and also in USGS ScienceBase repository (https://doi.org/10.5066/P9NLU6Q4):

Supplementary Dataset 1. *P. australis* version 1.0 reference gene models and syntelog pairs

Supplementary Dataset 2. Ortholog groups identified among *P. australis* and five monocots

Supplementary Dataset 3. GO clusters enriched among *P. australis* ortholog groups showing conserved or increased gene copy numbers due to *P. australis*-specific duplications

Supplementary Dataset 4. RNA-Seq raw read counts and expression rank percentiles for *P. australis* gene models for invasive and native genotypes.

Supplementary Dataset 5. RNA-Seq results comparing basal-level expression of *P. australis* genes among invasive and native genotypes

Supplementary Dataset 6. GO terms enriched among *P. australis* genes with significantly different basal-level expression among invasive and native genotypes.

Supplementary Dataset 7. RNA-Seq results for endophyte-responses of *P. australis* genotypes

Supplementary Dataset 8. GO terms enriched among *P. australis* genes whose expression were significantly induced or repressed by endophyte inoculation.
